# Dual regulation of bile acids: ameliorating hepatic glycolipid disorders and restoring intestinal health in HFHC-challenged Yellow River carp (*Cyprinus carpio* L.)

**DOI:** 10.1186/s12917-026-05688-1

**Published:** 2026-07-07

**Authors:** Xiao Yan, Bowen Yang, Jiali Mi, Shaoyang Zhi, Junchang Feng, Lijiao Cheng, Junli Wang, Chaobin Qin, Guoxing Nie

**Affiliations:** 1 College of Fisheries, Henan Normal University, No. 46 Jianshe Road, Xinxiang, 453007 P. R. China; 2 College of Life Sciences, Henan Normal University, No. 46 Jianshe Road, Xinxiang, 453007 P. R. China; 3 College of Fisheries, Xinyang Agriculture and Forestry University, Qianjin street, Xinyang, 464000 P. R. China

**Keywords:** Yellow River carp (*Cyprinus carpio* L.), Bile acids, Glycolipid metabolism, Gut microbiota, FXR, TGR5

## Abstract

**Background:**

This study was designed to investigate how three distinct bile acids (BAs) modulate glycolipid metabolic disorders and hepatointestinal injury induced by excessive intake of lipids and carbohydrates in Yellow River carp (*Cyprinus carpio* L.) and elucidate the underlying mechanisms involved. Here, the fish were randomly assigned to five groups: a control group (CON), a high-fat high-carbohydrate diet (HFHC) group, a HFHC + 300 mg/kg chenodeoxycholic acid (CDCA) group, a HFHC + 300 mg/kg ursodeoxycholic acid (UDCA) group and a HFHC + 300 mg/kg hyodeoxycholic acid (HDCA) group.

**Results:**

The results revealed that the serum triglyceride, glucose, and total cholesterol levels were significantly elevated in HFHC-fed fish, accompanied by increased glutamic-oxaloacetic transaminase (GOT) and glutamic-pyruvic transaminase (GPT) activities in the serum and hepatopancreas. However, dietary supplementation with bile acids in the HFHC diet significantly improved these negative changes. Analysis of BA-glycolipid metabolism-related gene expression and enzyme activities in the hepatopancreas revealed that CDCA and HDCA inhibited gluconeogenesis (FBPase/PEPCK/G6Pase) and lipogenesis (SREBP-1/FAS), while promoting glycogen accumulation (genes and glycogen levels) and fatty acid β-oxidation (PPARα) via activation of the FXR (farnesoid X receptor) /SHP (small heterodimer partner) pathway. In contrast, dietary UDCA supplementation increased intestinal TGR5 (takeda G protein-coupled receptor 5) expression and suppressed the activities of two key gluconeogenic enzymes, PEPCK and G6Pase. Additionally, dietary BAs supplementation alleviated HFHC diet-induced intestinal inflammation by inhibiting the NF-κB (Nuclear Factor κB) pathway. Bile acids relieved gut dysbiosis, improved microbial alpha diversity and community structure, and enriched beneficial bacteria including *Cetobacterium somerae*. These microbial changes eventually modulated host substance synthesis and metabolism. HE staining showed that HFHC diet caused hepatopancreatic lesions and intestinal morphological damage in Yellow River carp, which were effectively alleviated by bile acid addition.

**Conclusions:**

In conclusion, HFHC diets disrupt fish glycolipid metabolism and impair hepato-intestinal health in Yellow River carp, whereas dietary BAs can attenuate these detrimental effects by modulating metabolic pathways and the gut microbiota composition.

**Graphical Abstract:**

**Figure:**
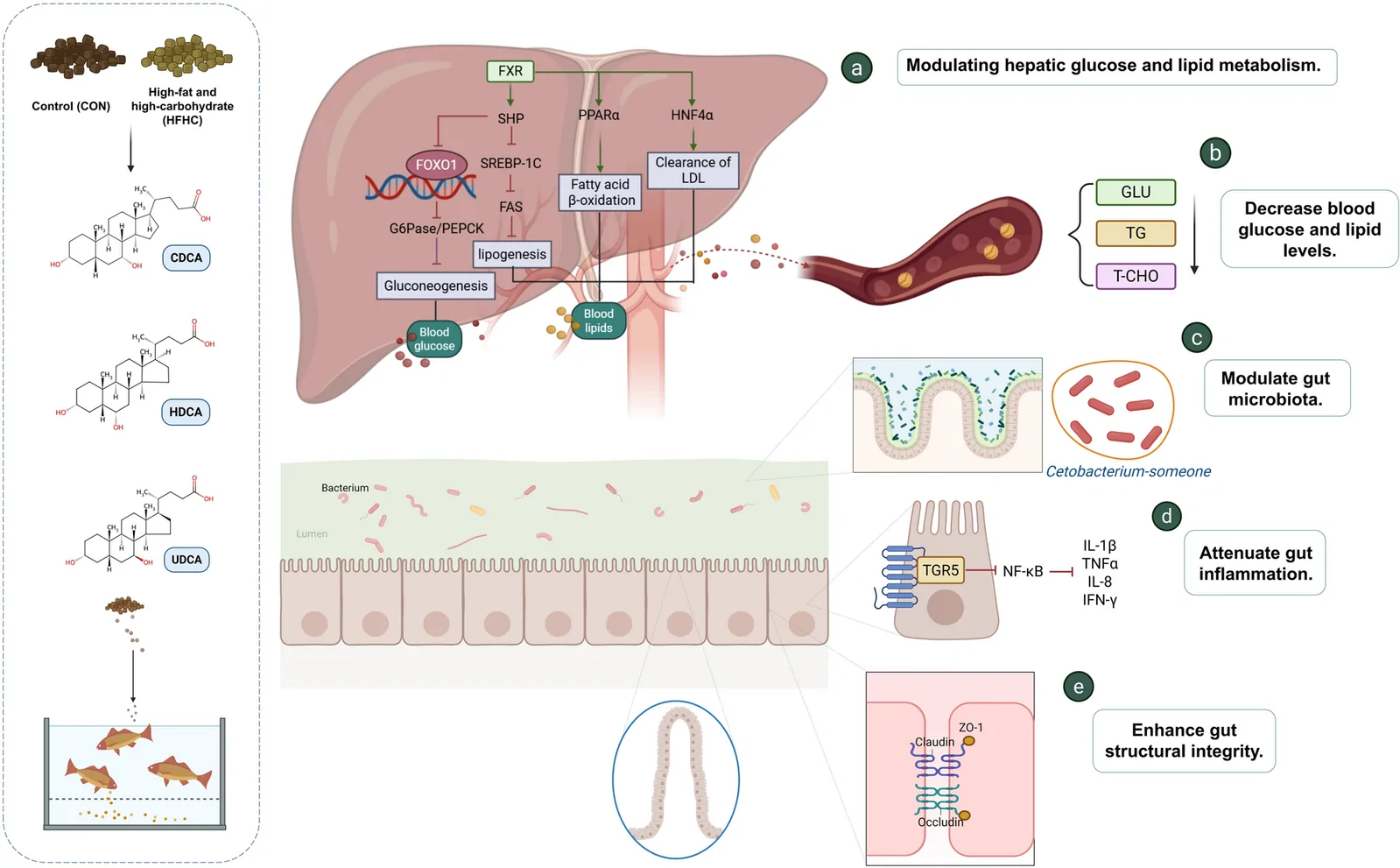

**Supplementary Information:**

The online version contains supplementary material available at https://doi.org/10.1186/s12917-026-05688-1.

## Introduction

In aquatic feed, carbohydrates and lipids function as economical energy sources but are crucial for fish developmental processes [1]. These macronutrients increase energy utilization efficiency in fish by optimizing metabolic pathways and energy allocation mechanisms, thereby promoting optimal growth and development [2–4]. Strategic incorporation of elevated proportions of carbohydrates and lipids in aquatic feed formulations not only effectively mitigates dependence on protein-based raw materials and reduces feed costs but also plays a positive role in decreasing ammonia nitrogen emissions in fish excreta and minimizing aquatic environment pollution [5–7]. However, excessive dietary carbohydrate and fat contents can decrease fish growth rates [8, 9], potentially precipitating metabolic disorders, hepatic steatosis, and compromised immune function [9, 10], all of which adversely affect the physiological health of fish. Consequently, investigating feed supplements capable of mitigating metabolic disruptions induced by high-fat and high-carbohydrate dietary regimens is essential for the sustainable development of aquaculture.

Bile acids (BAs) are a class of compounds synthesized from cholesterol via hepatic metabolic pathways. The biosynthesis of Bas starts with cholesterol and proceeds through multiple enzyme-catalyzed transformations in hepatocytes, after which the synthesized BAs are then stored in the gallbladder. During digestion, BAs are released into the small intestinal lumen, where they facilitate lipid emulsification, consequently enhancing lipid digestion and absorption [11]. BAs not only promote the digestion and absorption of lipids but also interact with the gut microbiota to influence the host’s metabolic and immune regulatory mechanisms [12]. Additionally, BAs play a crucial role in the regulation of glucose homeostasis [13]. These diverse functions highlight the pleiotropic roles of BAs in host physiological and pathological processes, with their complex metabolic pathways and regulatory mechanisms being the focus of current research [14].

As a key bile acid-sensing receptor, the farnesoid X receptor (FXR) exerts significant control over both bile acid homeostasis and glycolipid metabolic processes [15]. In hepatic tissues, FXR regulates bile acid biosynthesis by increasing the expression of small heterodimer partner (SHP) and inhibiting the transcriptional activity of both cholesterol 7α-hydroxylase (CYP7A1) and cholesterol 12α-hydroxylase (CYP8B1) [16, 17]. FXR activation in intestinal cells stimulates the release of fibroblast growth factor 15/19 (FGF15/19), which subsequently downregulates CYP7A1 expression, thereby exerting negative feedback on bile acid synthetic pathways [18, 19]. FXR modulates blood glucose levels by promoting fibroblast growth factor 19 (FGF19) secretion and downregulating the expression of cAMP response element-binding protein (CREB), G6Pase, and PEPCK [20, 21]. With respect to lipid metabolism regulation, FXR suppresses the transcriptional function of sterol regulatory element-binding protein (SREBP) by inducing SHP expression, consequently modulating hepatic lipogenesis [22]. Moreover, FXR enhances fatty acid catabolism while reducing lipid deposition by upregulating the expression of peroxisome proliferator-activated receptors (PPARs) [23].

Animals possess numerous structural variants of BAs within their biological systems. Chenodeoxycholic acid (CDCA) has been extensively shown to play pivotal regulatory roles in lipid homeostasis and immunomodulation [24, 25]. Ursodeoxycholic acid (UDCA) has been shown to facilitate bile secretion, increase nutrient digestion efficiency, improve hepatic function, and modulate lipid metabolism [26]. The scientific interest in hyodeoxycholic acid (HDCA) has increased because of its therapeutic prospects against atherosclerosis through its cholesterol-reducing capabilities [27]. In aquaculture, supplementation with specific BAs has been demonstrated to increase fish growth and mitigate lipid accumulation. The evidence suggests that dietary addition of CDCA accelerates growth performance while decreasing fat deposition in large yellow croaker specimens [28]. UDCA supplementation in juvenile large yellow croaker improved growth and lowered the feed conversion ratio [29]. Supplementation of high-fat diets with HDCA resulted in diminished hepatic lipid accumulation in tiger puffer [30]. The collective findings emphasize the potential of CDCA, UDCA, and HDCA as effective aquatic feed additives and support their further research and application.

This study used Yellow River carp as the research object, with the FXR signaling pathway as the entry point, and investigated the effects of CDCA, UDCA, and HDCA on glucose and lipid metabolism, as well as hepatic and intestinal health, in Yellow River carp under high-fat and high-carbohydrate (HFHC) dietary conditions. This study aims to lay a scientific foundation for optimizing the formulation of HFHC feeds for Yellow River carp farming and further promote the rational, safe, and economical utilization of feeds.

## Materials and methods

### Animal ethics statement

The Yellow River carp used in this study were purchased from a aquatic breeding farm in Yanjin, Henan Province, China. All experimental procedures involving animals adhered to ethical guidelines and were approved by the Institutional Animal Care and Use Committee of Henan Normal University (Approval No. HNSD-2026BS-0303).

### Animals and experiment design

A feeding trial lasting 56 days was conducted via pond cage cultivation methodology. The experiment involved 450 juvenile Yellow River carp with an average initial weight of 48.10 ± 0.01 g, which are randomly distributed across five experimental treatments, each comprising three replicates of 30 fish. The experimental diets were formulated with fish meal and corn gluten meal as the primary protein sources, wheat flour and corn starch as the principal carbohydrate sources, and soybean oil as the main lipid source. Five isonitrogenous experimental diets were used: control (CON), high-fat and high-carbohydrate (HFHC), and three additional formulations in which the HFHC diet was supplemented with 300 mg/kg of CDCA, UDCA, or HDCA. Table S1 provides comprehensive information regarding dietary formulations and their nutritional profiles. CDCA, UDCA, and HDCA were purchased from Aladdin Reagents Co. Ltd. (Shanghai, China). A dietary bile acid concentration of 300 mg/kg was used in this experiment, which was determined based on relevant domestic and international literature as well as our preliminary experimental results [31–33].

 a two-week acclimation period during which they were fed the control diet to facilitate adaptation to the experimental conditions. During the experiment, fish were fed three times daily at 07:00, 12:00 and 18:00, with the daily feeding amount accounting for 3.5% of the body weight of fish. The following environmental parameters were maintained: water temperature, approximately 26 °C; pH, approximately 7.0; dissolved oxygen. > 5.0 mg·L^− 1^; ammonia nitrogen, < 0.5 mg/L; and natural lighting.

### Sample collection and analysis

After the feeding trial, the experimental fish were weighed and measured. Three fish were subsequently randomly selected from each cage and anesthetized with 55 mg·L^− 1^ MS-222 (Aladdin, Shanghai, China) until all swimming movements ceased. The fish were then immediately euthanized via rapid spinal cord transection. Visceral weight, carcass weight, hepatopancreatic weight, and fat weight were recorded. Intact hepatopancreatic and intestinal tissues were preserved in 4% paraformaldehyde for histomorphological analysis. Portions of hepatopancreatic tissue and dorsal white muscle samples were used for proximate composition analysis. Samples of white muscle, intestine, and hepatopancreas were flash-frozen in liquid nitrogen and stored at -80℃ for subsequent analyses of gene expression, enzyme activities, and biochemical parameters. Blood samples were collected via tail vein phlebotomy, kept at 4 ℃ for more than 4 h, and centrifuged at 3500 rpm for 10 min at 4 ℃. The obtained serum was collected for subsequent physiological and biochemical analyses. Intestinal samples were rapidly dissected under aseptic conditions. The intestinal contents of each fish were squeezed into 10 mL nuclease-free sterile centrifuge tubes and immediately stored at -80 °C for subsequent third-generation 16 S rRNA sequencing.

### Growth performance analysis

Growth metrics, dietary efficiency parameters, and visceral characteristics were assessed through the application of standardized mathematical equations. Additionally, the survival rate (SR) was determined. The following indicators were evaluated: initial body weight (IBW), final body weight (FBW), weight gain rate (WGR), specific growth rate (SGR), condition factor (CF), feed efficiency (FE), visceral index (VSI), and hepatic somatic index (HSI). The calculation formula is as follows:

$$\begin{aligned} \mathrm{SR}\left(\%\right)&=\text{number of surviving experimental fish}\\&/\text{total number of experimental fish}\times{100} \end{aligned}$$

$$\mathrm{WGR}\left(\%\right)=\left(\mathrm{FBW-IBW}\right)/\mathrm{IBW}\times{100}$$

$$\begin{aligned} \mathrm{SGR}\left(\%/\mathrm{d}\right)&=\left(\text{Ln FBW-Ln IBW}\right)\\&/\text{breeding days}\times{100} \end{aligned}$$

$$\mathrm{CF}\left(\mathrm{g/cm}^{3}\right)=\text{FBW/body length}^{3}\times{100}$$

$$\mathrm{FE}\left(\%\right)=\left(\mathrm{FBW-IBW}\right)/\text{Food intake}\times{100}$$

$$\mathrm{VSI}\left(\%\right)=\text{wet weight of viscera/FBW}\times{100}$$

$$\mathrm{HSI}\left(\%\right)=\text{wet weight of hepatopancreas/FBW}\times{100}$$

### Proximate composition analysis

The approximate compositions of the diet, hepatopancreas, and muscle were determined according to standard methods [34]. The samples were first ground into a powder, weighed, and then placed in an oven at 105 °C for drying until a constant weight was attained. The moisture content of the samples was calculated on the basis of the weight difference before and after drying.

All the samples were placed in crucibles and carbonized on a heating mantle.The carbonized samples were subsequently transferred to a muffle furnace and ignited to ash at 550 °C. After ashing, the samples were moved to a desiccator for cooling and weighing to determine the crude ash content.

The crude protein content was assayed via the Kjeldahl method. This approach estimates protein content by quantifying the nitrogen content in samples. Specifically, the procedure involved digesting the sample to convert nitrogen-containing components into ammonium sulfate; ammonia gas was then released via distillation under alkaline conditions. Ammonia was then titrated, from which the crude protein content of the sample was calculated.

The crude lipid content was determined following the Soxhlet extraction method. The ground samples were placed in a Soxhlet extractor with petroleum ether as the extraction solvent, and the lipids in the samples were separated via solvent extraction. The extracted lipids were subsequently dried to a constant weight and subjected to gravimetric analysis.

### Serum and tissue biochemical parameter measurements

Circulating concentrations of glucose (GLU), triglycerides (TG), total cholesterol (T-CHO), hydrogen peroxide (H_2_O_2_), and malondialdehyde (MDA) were assessed in blood serum, while the cholesterol fractions (T-CHO, high-density lipoprotein cholesterol (HDL-C), low-density lipoprotein cholesterol (LDL-C)) and the oxidative stress indicator (MDA) were analyzed in hepatopancreatic tissue. The above indicators were analyzed via commercial assay kits manufactured by Nanjing Jiancheng Bioengineering Institute.

### Serum and tissue enzyme activity measurements

The enzymatic activities of transaminases (GPT and GOT) were evaluated in both serum and hepatopancreatic tissues, whereas the activities of antioxidant enzymes (superoxide dismutase (T-SOD)) were assessed exclusively in the hepatopancreas via diagnostic reagents supplied by Nanjing Jiancheng Bioengineering Institute, following established protocols. The activities of glycolytic and gluconeogenic enzymes (hexokinase (HK), pyruvate kinase (PK) and phosphoenolpyruvate carboxykinase (PEPCK) in hepatopancreatic extracts were quantified via commercial assay kits from Beijing Solarbio, with methodologies adhering to the manufacturer’s specifications.

### Histological analysis

Following immersion of the intestinal and hepatopancreatic tissues in 4% paraformaldehyde solution, residual fixative was eliminated through sequential buffer washes. The tissue samples were subsequently subjected to a graduated ethanol dehydration series (75%, 80%, 90%, 95%, and 100% ethanol), followed by xylene-mediated transparency and paraffin embedding. Sections were prepared via a microtome, stained with hematoxylin and eosin (H&E), and examined under an optical microscope [34].

### PAS staining of glycogen in the hepatopancreas

Hepatopancreas sections were prepared from paraffin-embedded samples. The tissue sections underwent xylene dewaxing, followed by gradual rehydration with ethanol, periodate solution oxidation, and dark incubation with Schiff reagent for staining. After rinsing with water, the sections were counterstained with hematoxylin and blued with bluing buffer. Finally, the sections were dehydrated with anhydrous ethanol, cleared with toluene, and mounted with neutral gum.

### Hepatopancreas immunofluorescence

Hepatopancreas samples were embedded in paraffin, sectioned, and mounted on slides. Xylene was used for section dewaxing, while a graded ethanol series was used for dehydration. The sections were subsequently incubated with 3% H_2_O_2_ in the dark to inhibit endogenous peroxidase activity. The sections were washed with PBS, followed by blocking with goat serum. They were then incubated with primary antibody and subsequently with secondary antibody, with both processes conducted under dark conditions. The sections were then counterstained with 4’,6-diamidino-2-phenylindole (DAPI) and mounted with anti-fade mounting medium. Fluorescence images were captured by confocal microscopy through the fluorescein isothiocyanate (FITC) and DAPI channels, while the fluorescence intensity was quantitatively analyzed via ImageJ software. Primary antibodies against FXR (Cat# bs-12867R), SHP (Cat# bs-4311R), FOXO1A (Cat# bs-9439R), and G6Pase (Cat# bs-4044R) were obtained from Bioss Inc. DAPI staining solution (Cat# G1012) obtained from Servicebio Inc.

### Determination of glycogen content in hepatopancreatic tissue

The glycogen content in hepatopancreatic tissue was detected via commercially available assay kits (Beijing Solarbio).

### Quantitative real-time PCR

Total RNA was extracted from hepatopancreas and intestinal samples via Trizol reagent obtained from Takara, Japan. A NanoDrop 2000 C spectrophotometer was used to quantify the RNA concentration, and the RNA integrity was assessed via agarose gel electrophoretic analysis. The isolated total RNA was reverse transcribed into cDNA via kits provided by the same manufacturer as mentioned previously. For real-time quantitative PCR (qPCR), the reaction mixture consisted of 10 µL total volume (0.6 µL of primer, 0.1 µL of cDNA, 5 µL of qPCR SuperMix, and 4.3 µL of RNA-free water). Table S2 presents the comprehensive primer sequences utilized for amplification of each target gene in this study.

### Gut microbiota analysis

The 16 S rRNA gene sequencing analysis of the fish intestinal content samples was performed by Shanghai Personal Biotechnology Co., Ltd. Total DNA was extracted via the OMEGA soil DNA Kit (Omega Bio-Tek, USA), and the V1‒V9 hypervariable regions of the 16 S rRNA gene were amplified via PCR. Prior to SMRT sequencing procedures, purification and quantification of the PCR amplicons were accomplished with a Quant-iT PicoGreen dsDNA Assay Kit supplied by Invitrogen (USA). The sequencing data were processed via QIIME 2 (2019.4) and DADA2 to obtain high-quality ASV sequences, which were annotated for taxonomy on the basis of the Greengenes database. Microbial community diversity analysis encompassed both alpha and beta diversity metrics through the QIIME 2 and R programming environments. The classification outcomes were visualized across taxonomic hierarchies, including the phylum, genus, and species levels. Additionally, principal coordinate analysis (PCoA) was conducted via R language and vegan software, and metabolic pathways were analyzed via the KEGG database.

### Statistical analysis and visualization

SPSS software (version 26.0) was used as the analytical platform for the statistical processing of the experimental data in this study. All the data are expressed as the mean ± standard error of the mean (S.E.M.). The independent samples t-test was used to evaluate significant differences between the control group and the HFHC group. To assess variations among multiple HFHC treatment regimens, one-way analysis of variance (ANOVA) was performed, followed by Tukey’s multiple comparison test for post-hoc analysis. A value of *P* < 0.05 was considered statistically significant. GraphPad Prism software (Version 10.0) was used for data visualization.

## Results

### Growth performance

The survival rate of the experimental fish was 100% following the feeding trial (Table 1). Compared with the CON treatment, the HFHC treatment notably increased the FBW, WGR, and SGR values (*P* < 0.05). Compared with the HFHC group, the UDCA group and HDCA group presented significantly better outcomes in terms of FBW, WGR, SGR, and FE (*P* < 0.05). The performance metrics of the fish receiving CDCA were statistically indistinguishable from those receiving HFHC.

**Table 1:** Growth performance of Yellow River carp in different experimental groups

Index	Groups				
	CON	HFHC	CDCA	UDCA	HDCA
IBW (g)	48.12 ± 0.05	48.13 ± 0.04	48.08 ± 0.03	48.10 ± 0.04	48.10 ± 0.01
FBW (g)	152.47 ± 1.07	179.47 ± 1.18^*b^	180.86 ± 0.71^b^	188.64 ± 2.24^a^	190.42 ± 1.44^a^
WGR (%)	216.89 ± 2.08	272.91 ± 2.17^*b^	276.20 ± 1.38^b^	292.21 ± 4.51^a^	295.90 ± 3.30^a^
SGR (%/d)	2.06 ± 0.01	2.35 ± 0.01^*b^	2.37 ± 0.00^b^	2.44 ± 0.02^a^	2.45 ± 0.01^a^
SR (%)	100 ± 0.00	100 ± 0.00	100 ± 0.00	100 ± 0.00	100 ± 0.00
FE (%)	66.10 ± 0.66	83.19 ± 0.72^*b^	84.10 ± 0.44^b^	89.01 ± 1.40^a^	90.14 ± 0.94^a^
CF (g/cm^3^)	2.45 ± 0.01	2.42 ± 0.02^a^	2.42 ± 0.02^a^	2.30 ± 0.02^b^	2.35 ± 0.02^b^
VSI (%)	8.04 ± 0.62	9.01 ± 0.18	9.28 ± 0.30	8.73 ± 0.38	8.97 ± 0.48
HSI (%)	1.62 ± 0.08	1.53 ± 0.06	1.45 ± 0.10	1.51 ± 0.07	1.64 ± 0.06

Data were presented as means ± S.E.M. (*n* = 3)

Different letters indicated significant difference between HFHC and BA groups (*P* < 0.05). ANOVA was performed, followed by Tukey’s multiple comparison test for post-hoc analysis

^*^indicated significant difference between CON and HFHC groups (*P* < 0.05), The independent samples t-test was used to evaluate statistical differences

### Proximate composition analysis

The nutritional compositions of the muscle and hepatopancreas tissues in the different experimental groups are presented in Table 2.

**Table 2:** Composition of conventional components of Yellow River carp muscle and hepatopancreas in different experimental groups

Index	Groups				
	CON	HFHC	CDCA	UDCA	HDCA
Muscle					
Moisture (%)	78.73 ± 0.11	76.54 ± 0.21^*b^	77.35 ± 0.80^ab^	77.70 ± 0.20^a^	77.72 ± 0.47^a^
Crude lipid (%/WM)	5.34 ± 0.23	11.40 ± 0.25^*a^	11.01 ± 0.24^a^	8.78 ± 0.61^b^	10.39 ± 0.47^a^
Crude protein (%/WM)	18.89 ± 0.40	18.88 ± 0.34^b^	21.11 ± 0.26^a^	21.45 ± 0.54^a^	21.40 ± 0.25^a^
Hepatopancreas					
Moisture (%)	69.15 ± 0.33	64.58 ± 1.52^*b^	68.21 ± 1.68^ab^	69.75 ± 0.01^a^	70.71 ± 0.59^a^
Crude lipid (%/WM)	1.74 ± 0.01	3.05 ± 0.22^*a^	2.13 ± 0.02^b^	2.50 ± 0.32^ab^	1.70 ± 0.22^b^
Crude protein (%/WM)	21.35 ± 0.01	13.78 ± 0.03^*c^	15.65 ± 0.01^a^	14.58 ± 0.03^b^	13.55 ± 0.02^d^

Data were presented as means ± S.E.M. (*n* = 3)

Different letters indicated significant difference between HFHC and BA groups (*P* < 0.05). ANOVA was performed, followed by Tukey’s multiple comparison test for post-hoc analysis

^*^indicated significant difference between CON and HFHC groups (*P* < 0.05), The independent samples t-test was used to evaluate statistical differences

Compared with the CON diet, the HFHC diet significantly decreased the moisture content and significantly increased the crude lipid content in muscle (*P* < 0.05). Compared with those in the HFHC group, the muscle moisture and crude protein contents in the UDCA and HDCA treatment groups significantly increased (*P* < 0.05), whereas the UDCA group presented significantly lower crude lipid contents than did the HFHC group (*P* < 0.05).

Compared with the CON group, the HFHC group presented a significantly greater crude lipid content and significantly lower moisture and crude protein contents in the hepatopancreas (*P* < 0.05). Compared with the HFHC group, the UDCA and HDCA treatment groups presented significantly greater hepatopancreatic moisture contents (*P* < 0.05). The CDCA and HDCA supplementation groups presented significantly lower crude lipid contents than did the HFHC group (*P* < 0.05). In addition, the crude protein content was significantly greater in the CDCA and UDCA groups than in the HFHC group (*P* < 0.05).

### Serum and hepatopancreas biochemical parameters

**Fig. 1:**
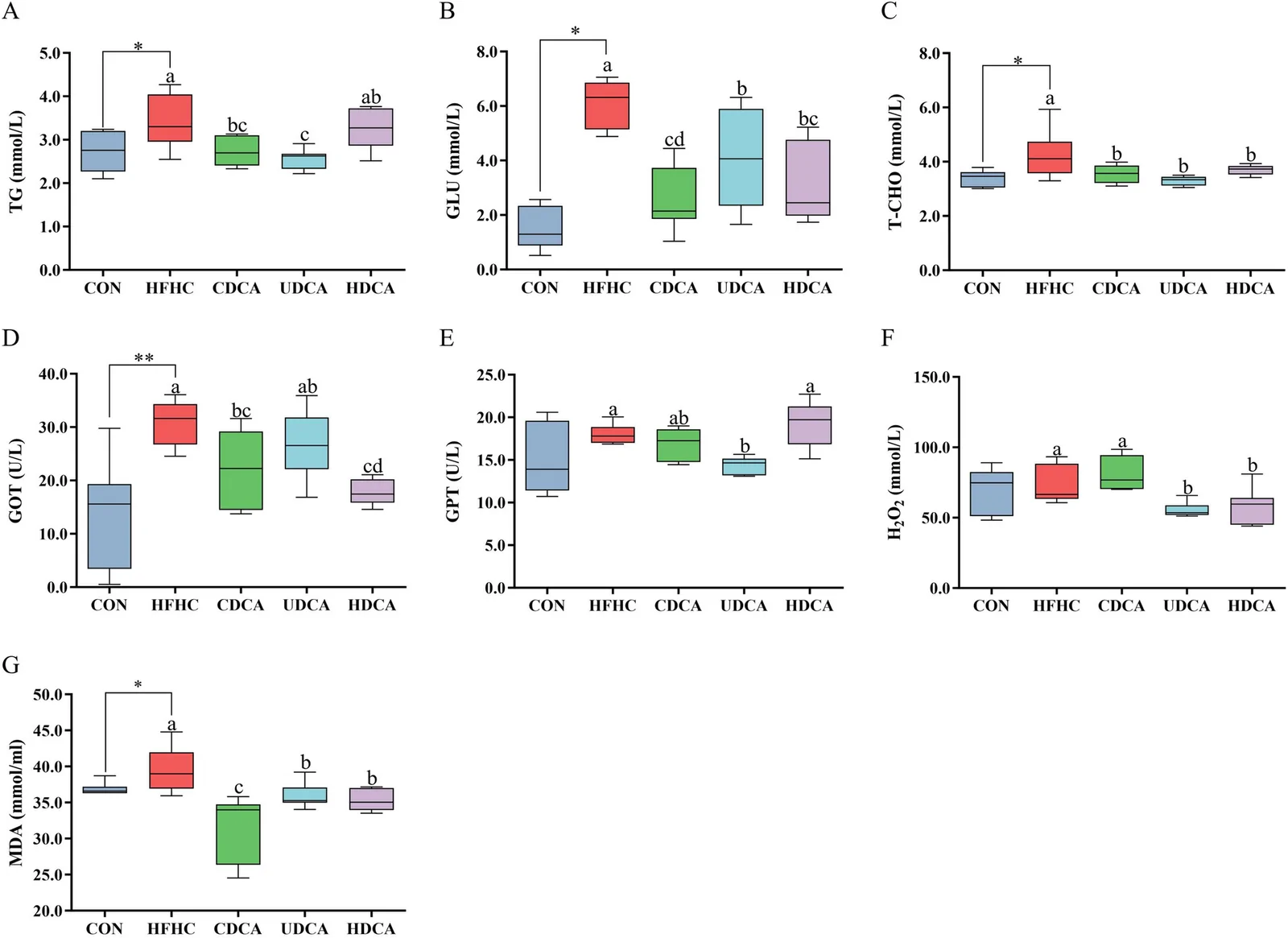
Effect of BAs on serum physiological, biochemical and antioxidant parameters in Yellow River carp. **A** TG, **B** GLU, **C** T-CHO, **D** GOT, **E** GPT, **F** H_2_O_2_, **G** MDA. Data are presented as means ± S.E.M. The “*” indicates a significant difference between the control group and the HFHC group (*P* < 0.05). Different letters indicate significant differences between the HFHC group and the BAs group (*P* < 0.05)

Serum analysis revealed that, compared with the control fish, the HFHC diet-fed fish presented significantly elevated serum TG concentrations (*P* < 0.05). Supplementation with CDCA and UDCA markedly reduced the serum TG levels (*P* < 0.05, Fig. 1A). Furthermore, compared with the control fish, the HFHC diet-fed fish presented significantly higher serum GLU and T-CHO levels (*P* < 0.05). In contrast, the administration of all the tested bile acid compounds effectively lowered the serum GLU and T-CHO levels (*P* < 0.05, Fig. 1B and C). In terms of aminotransferase activity, the GOT activity in the HFHC group was significantly greater than that in the control group (*P* < 0.05), whereas CDCA and HDCA supplementation significantly reduced GOT activity (*P* < 0.05, Fig. 1D). GPT activity was significantly decreased following UDCA supplementation (*P* < 0.05, Fig. 1E). The serum H_2_O_2_ concentration was significantly lower in the UDCA and HDCA groups than in the HFHC group (*P* < 0.05, Fig. 1F). Compared with those in the HFHC group, the serum MDA levels of the fish in the bile acid-supplemented diets were significantly lower, which indicated alleviated oxidative stress (*P* < 0.05, Fig. 1G).

**Fig. 2:**
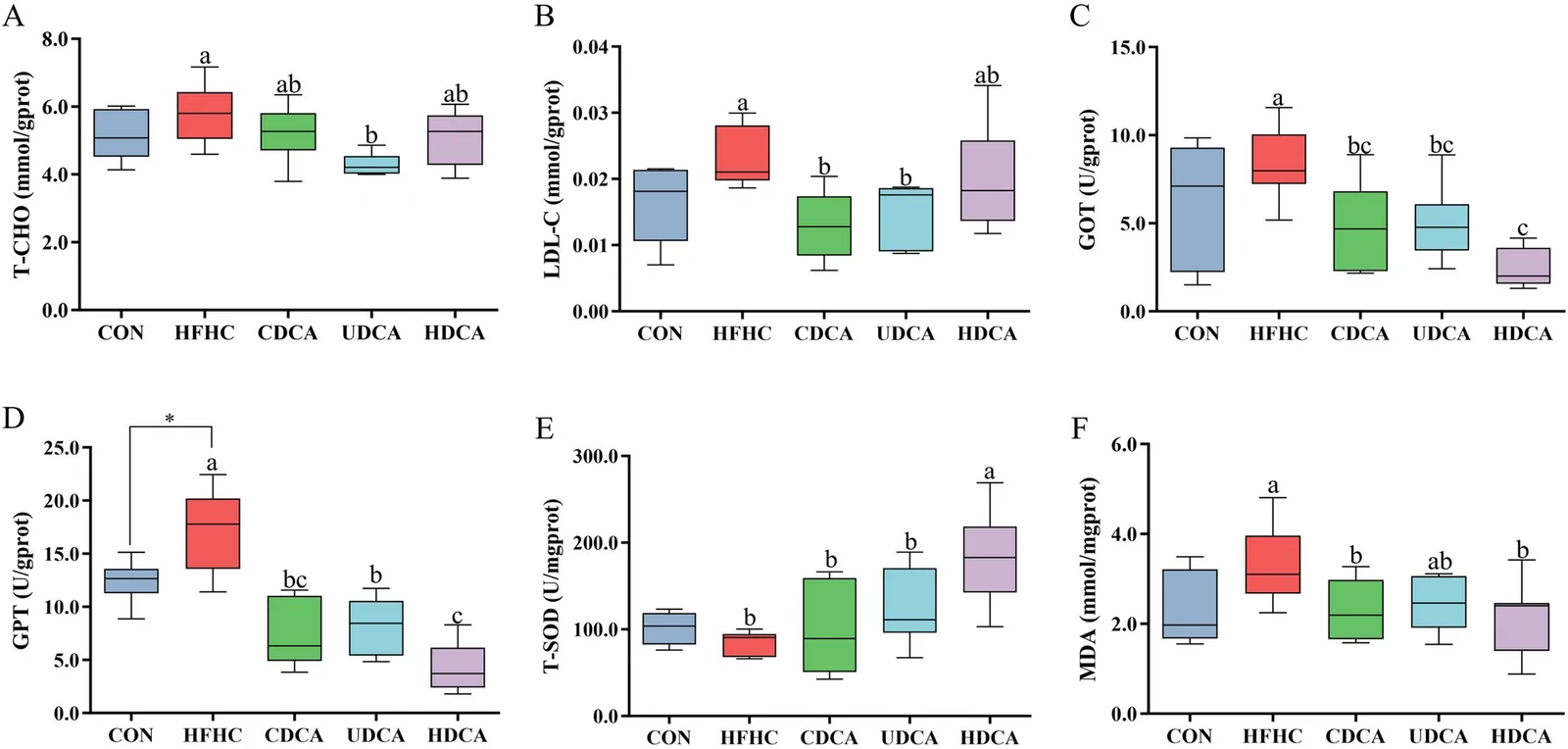
Effect of BAs on hepatopancreas physiological, biochemical parameters, and antioxidant parameters in Yellow River carp. **A** T-CHO, **B** LDL-C, **C** GOT, **D** GPT, **E** T-SOD, **F** MDA. Data are presented as means ± S.E.M. The “*” indicates a significant difference between the control group and the HFHC group (*P* < 0.05). Different letters indicate significant differences between the HFHC group and the BAs group (*P* < 0.05)

The biochemical parameters of the hepatopancreas are presented in Fig. 2. Compared with those in HFHC-fed fish, hepatopancreatic tissue in fish supplemented with UDCA presented significantly lower T-CHO levels (*P* < 0.05, Fig. 2A). Additionally, CDCA and UDCA supplementation significantly decreased hepatopancreatic LDL-C concentrations (*P* < 0.05, Fig. 2B). Enzymatic assays revealed that hepatopancreatic GPT activity was significantly greater in the HFHC group than in both the control and bile acid-supplemented groups (*P* < 0.05, Fig. 2D). Hepatopancreatic T-SOD activity was not significantly different between the HFHC and control groups, whereas HDCA supplementation significantly increased the activity of this antioxidant enzyme compared with that in the HFHC group (*P* < 0.05, Fig. 2E). Furthermore, the hepatopancreatic MDA content was significantly lower in the CDCA and HDCA groups than in the HFHC group (*P* < 0.05, Fig. 2F).

### Hepatopancreas glucose metabolizing enzyme activity

**Fig. 3:**
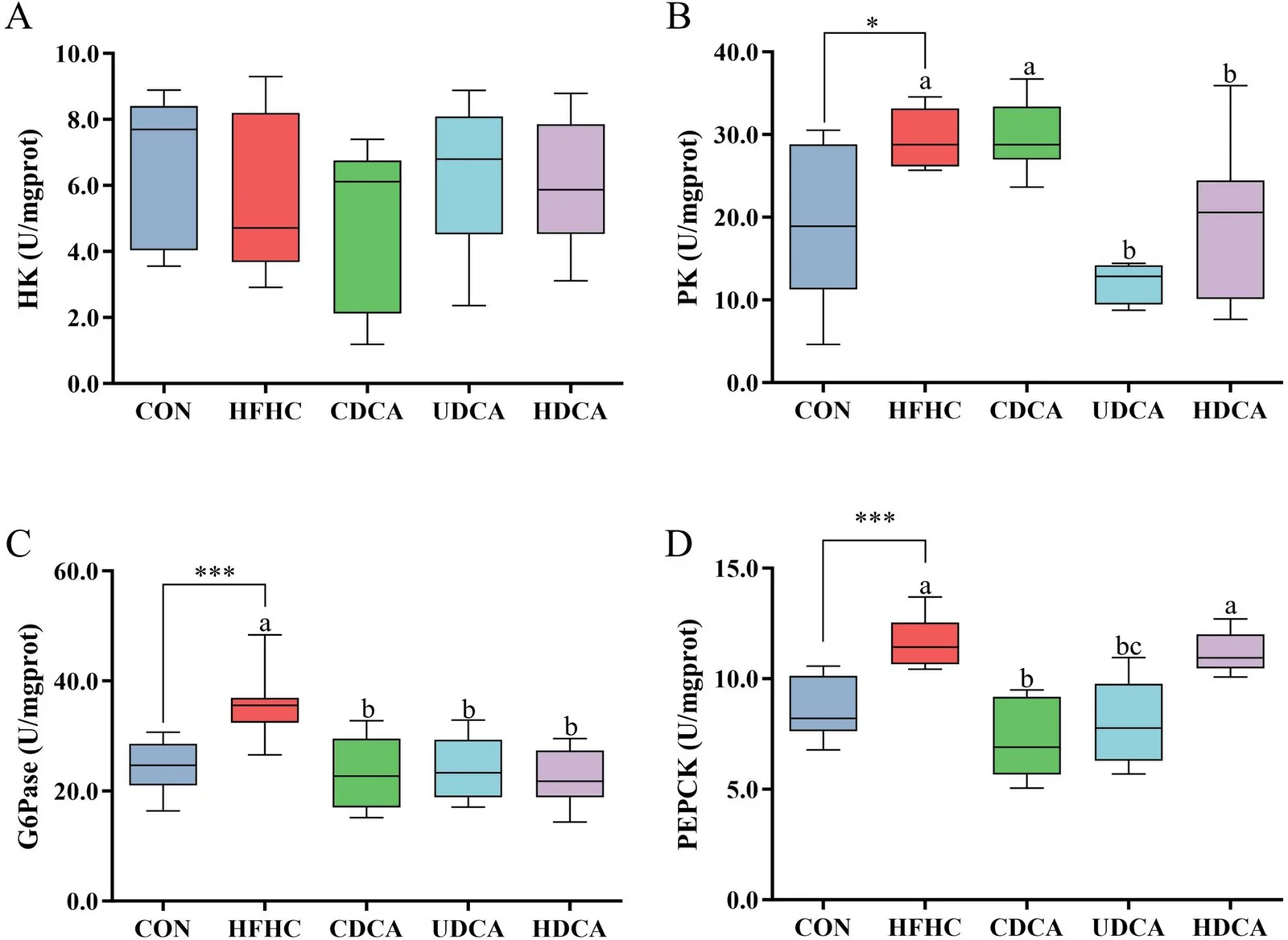
Effect of BAs on the activities of glucose metabolism-related enzymes in the hepatopancreas. **A** HK, **B** PK, **C** G6Pase, **D** PEPCK. Data are presented as means ± S.E.M. The “*” indicates a significant difference between the control group and the HFHC group (*P* < 0.05). Different letters indicate significant differences between the HFHC group and the BAs group (*P* < 0.05)

Figure 3 shows the glucose-related enzyme activities in the hepatopancreatic tissue of Yellow River carp. With respect to the glycolytic pathways, hexokinase (HK) activity did not significantly differ across all the experimental groups (Fig. 3A). In contrast, fish fed the HFHC diet presented markedly elevated pyruvate kinase (PK) activity compared with those in the control, UDCA, and HDCA groups (*P* < 0.05, Fig. 3B). In terms of the gluconeogenic pathways, HFHC diet feeding significantly increased the activities of both G6Pase and PEPCK relative to those in the control group (*P* < 0.05, Fig. 3C and D). Compared with the HFHC diet, bile acid supplementation effectively reduced G6Pase activity (*P* < 0.05, Fig. 3C), whereas CDCA and UDCA supplementation significantly downregulated PEPCK activity (*P* < 0.05, Fig. 3D).

### Gene expression related to bile acid-glycolipid metabolism in the hepatopancreas

**Fig. 4:**
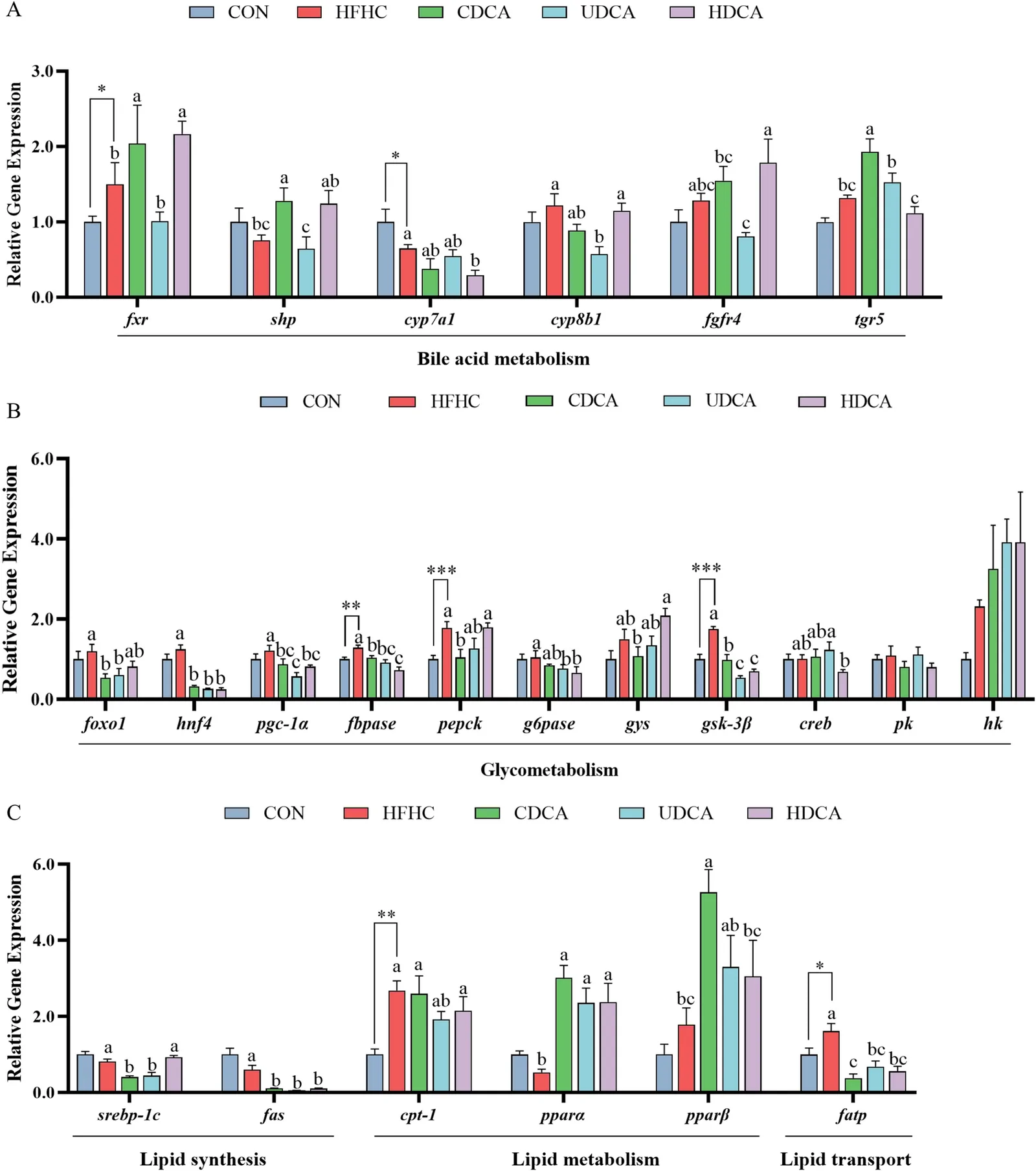
The effects of BAs on the genes expression in the hepatopancreas. **A** bile acid metabolism, **B** glycometabolism, **C** lipid metabolism. Data are presented as means ± S.E.M. The “*” indicates a significant difference between the control group and the HFHC group (*P* < 0.05). Different letters indicate significant differences between the HFHC group and the BAs group (*P* < 0.05)

The mRNA expression levels of genes related to bile acid metabolism in the hepatopancreas are presented in Fig. 4A. Compared with that in the control (CON) group, *fxr* mRNA expression in the HFHC group was significantly upregulated (*P* < 0.05), and further elevated following supplementation with CDCA and HDCA (*P* < 0.05). The *shp* mRNA levels in the CDCA group were significantly greater than those in the HFHC group (*P* < 0.05). Compared with that in the CON group, *cyp7a1* mRNA expression was significantly lower in the HFHC group (*P* < 0.05). Compared with the HFHC diet, HDCA supplementation further significantly decreased *cyp7a1* mRNA expression (*P* < 0.05). The *cyp8b1* mRNA levels in the UDCA group were significantly lower than those in the HFHC group (*P* < 0.05). Additionally, *tgr5* mRNA expression in the CDCA group was significantly greater than that in the HFHC group (*P* < 0.05).

The expression of genes related to glucose metabolism in the hepatopancreas is presented in Fig. 4B. Compared with the CON group, the HFHC group presented significantly upregulated mRNA expression of *fbpase*, *pepck*, and *gsk-3β* (*P* < 0.05). Compared with the HFHC diet, all three bile acid treatments significantly downregulated the mRNA expression of *foxo1*, *hnf4*, and *pgc-1α* (*P* < 0.05), although the regulatory magnitudes differed among the three BAs. Compared with the HFHC diet, all three bile acid treatments significantly decreased *fbpase* mRNA expression (*P* < 0.05). In addition, CDCA supplementation significantly downregulated *pepck* mRNA expression relative to that in the HFHC group (*P* < 0.05), whereas UDCA and HDCA supplementation significantly reduced *g6pase* mRNA expression (*P* < 0.05). Furthermore, all bile acid treatments significantly downregulated *gsk-3β* mRNA expression compared with that in the HFHC group (*P* < 0.05).

The expression of genes related to lipid metabolism in the hepatopancreas is presented in Fig. 4C. Compared with those in the CON group, there were no significant differences in *srebp-1c* or *fas* mRNA expression in the HFHC group. Compared with that in the HFHC group, *srebp-1c* mRNA expression was significantly lower in the CDCA and UDCA groups (*P* < 0.05), while all bile acid treatments significantly decreased *fas* mRNA expression (*P* < 0.05). Compared with the CON group, the HFHC group presented significantly upregulated *cpt-1* mRNA expression (*P* < 0.05). Furthermore, *pparα* mRNA expression was significantly greater in all three bile acid groups than in the HFHC group (*P* < 0.05), whereas the CDCA group presented significantly greater *pparβ* mRNA expression than did the HFHC group (*P* < 0.05). Compared with CON, HFHC diet feeding significantly upregulated *fatp* mRNA expression (*P* < 0.05), while each bile acid treatment reduced *fatp* transcription relative to that in the HFHC group (*P* < 0.05).

### Gene expression related to bile acid-glucose metabolism in the intestine

**Fig. 5:**
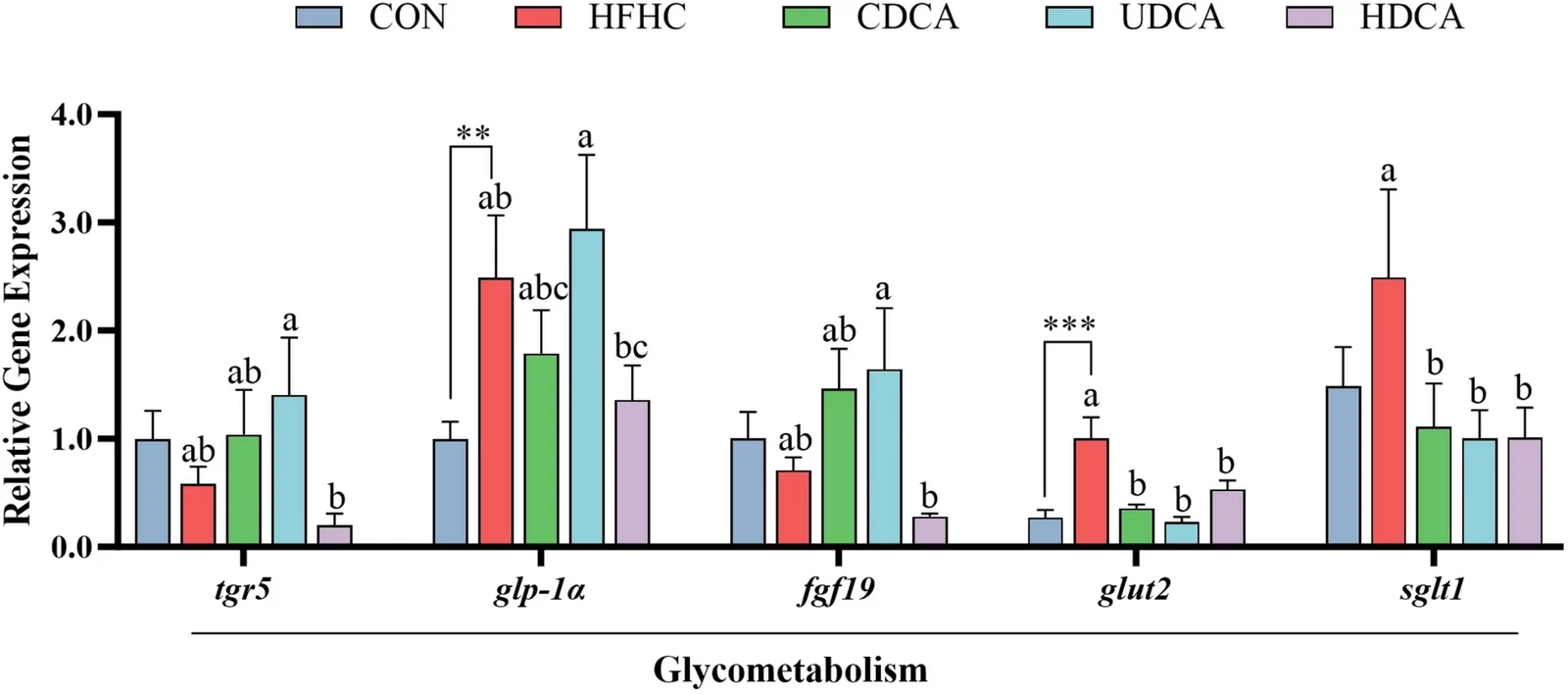
The effects of BAs on the expression levels of genes related to glycometabolism in the intestine of Yellow River carp. Data are presented as means ± S.E.M. The “*” indicates a significant difference between the control group and the HFHC group (*P* < 0.05). Different letters indicate significant differences between the HFHC group and the BAs group (*P* < 0.05)

Gene expression related to bile acid and glucose metabolism in the intestine is presented in Fig. 5. In terms of bile acid metabolism, UDCA supplementation significantly upregulated *tgr5* and *fgf19* mRNA expression compared with that in the HFHC group (*P* < 0.05), whereas HDCA supplementation downregulated their expression (*P* < 0.05). For glucose metabolism, *glp-1α* and *glut2* mRNA expression in the HFHC group was significantly greater than that in the CON group (*P* < 0.05). Compared with the HFHC diet, all bile acid treatments significantly suppressed *glut2* and *sglt1* mRNA expression (*P* < 0.05).

### Hepatopancreas immunofluorescence staining

**Fig. 6:**
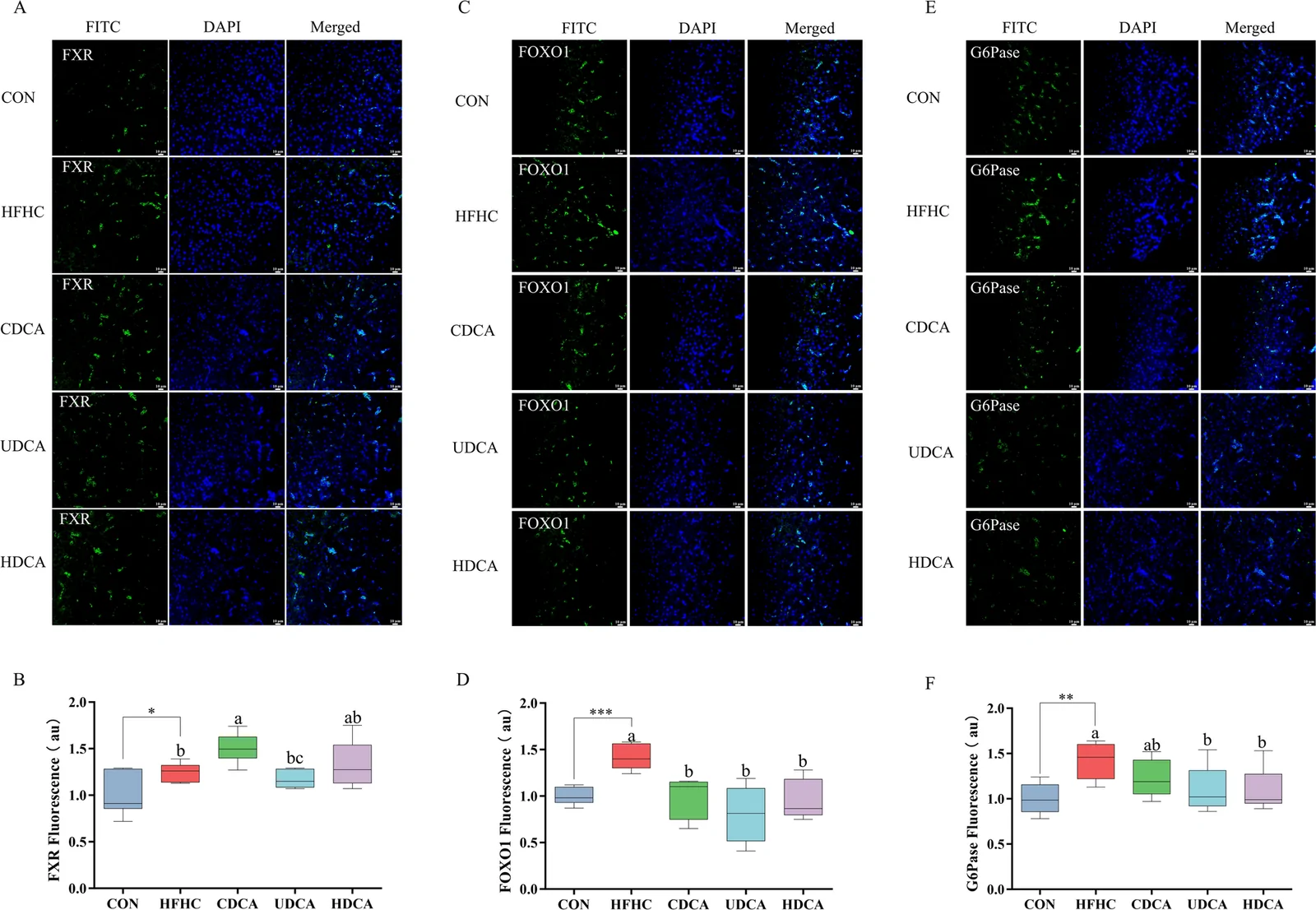
Immunofluorescence staining and quantitative results of FXR, FOXO1 and G6Pase protein in the hepatopancreas of Yellow River carp. **A**,** B** FXR, **C**,** D** FOXO1, **E**,** F** G6Pase. Data are presented as means ± S.E.M. The “*” indicates a significant difference between the control group and the HFHC group (*P* < 0.05). Different letters indicate significant differences between the HFHC group and the BAs group (*P* < 0.05)

Immunofluorescence staining was performed on hepatopancreas sections to detect the protein expression of FXR, FOXO1, and G6Pase. The target proteins were visualized with Alexa Fluor 488-conjugated secondary antibodies (green fluorescence), and the nuclei were counterstained with 4’,6-diamidino-2-phenylindole (DAPI, blue fluorescence). Representative immunofluorescence images and the quantitative fluorescence intensity data are presented in Fig. 6.

Compared with those in the CON group, the protein levels of FXR, FOXO1, and G6Pase in the HFHC group were significantly greater (*P* < 0.05). Compared with the HFHC diet, CDCA supplementation further significantly upregulated FXR protein expression (*P* < 0.05, Fig. 6A, B). All bile acid treatments significantly suppressed FOXO1 protein expression (*P* < 0.05, Fig. 6C, D). Moreover, both UDCA and HDCA significantly decreased G6Pase protein expression compared with that in the HFHC group (*P* < 0.05, Fig. 6E, F).

### Glycogen content and glycogen PAS staining

Glycogen levels in the hepatopancreas were quantified to assess metabolic changes. Compared with the control group, the HFHC group presented significantly higher hepatopancreatic glycogen levels (*P* < 0.05). Furthermore, both the hepatopancreas glycogen contents in the UDCA and HDCA groups were significantly elevated relative to those in the HFHC group (*P* < 0.05, Table 3).

**Table 3:** Effects of BAs on hepatopancreas glycogen content of Yellow River carp in different experimental groups

Index	Groups				
	CON	HFHC	CDCA	UDCA	HDCA
Glycogen (mg/g)	0.83 ± 0.01	0.99 ± 0.04^*b^	0.96 ± 0.03^b^	1.18 ± 0.06^a^	1.15 ± 0.04^a^

Data were presented as means ± S.E.M. (*n* = 3)

Different letters indicated significant difference between HFHC and BA groups (*P* < 0.05). ANOVA was performed, followed by Tukey’s multiple comparison test for post-hoc analysis

^*^indicated significant difference between CON and HFHC groups (*P* < 0.05), The independent samples t-test was used to evaluate statistical differences

**Fig. 7:**
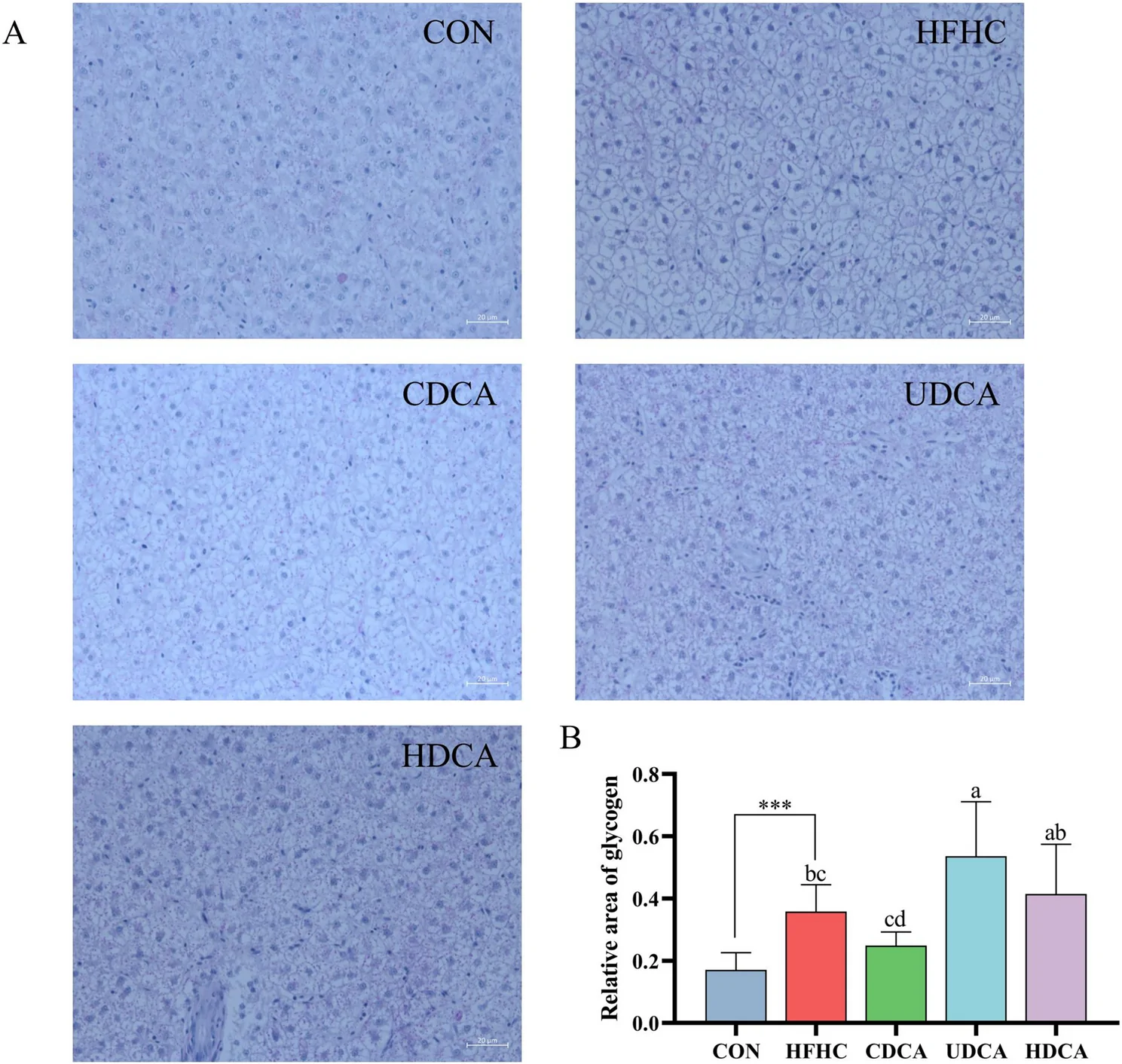
Hepatopancreas glycogen PAS staining and quantitative analysis of Yellow River carp. (**A)** PAS staining, (**B)** Quantitative analysis of glycogen content

Compared with the control diet, quantitative analysis of hepatopancreas PAS staining revealed that the HFHC diet significantly promoted glycogen accumulation (*P* < 0.05). Additionally, the glycogen content in both the UDCA and HDCA groups was significantly greater than that in the HFHC group (*P* < 0.05, Fig. 7).

### Hepatopancreas histomorphology

**Fig. 8:**
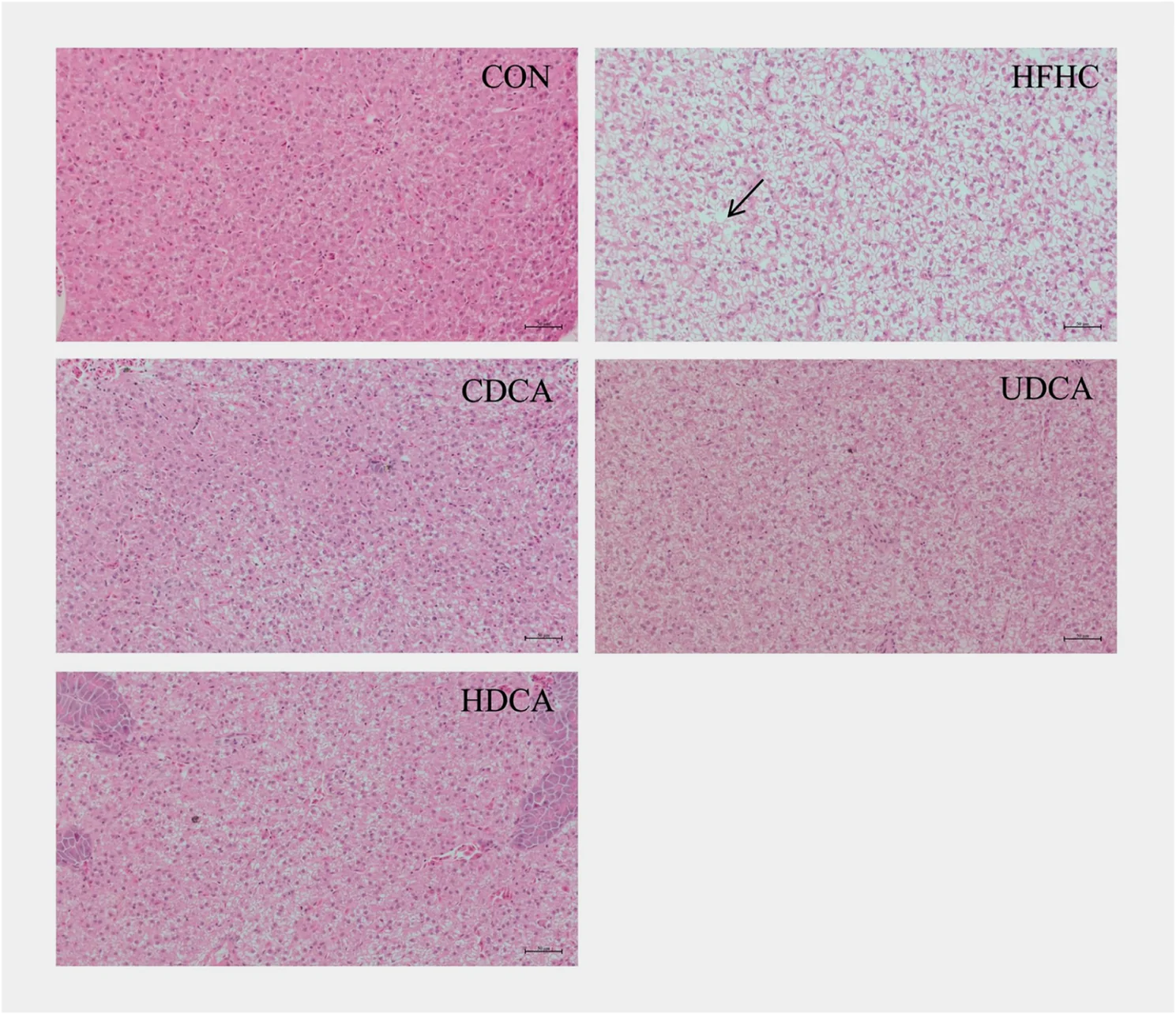
Hepatopancreas H&E staining of Yellow River carp

Hematoxylin‒eosin (H&E) staining was conducted on hepatopancreatic tissue sections, and representative micrographs are presented in Fig. 8. Compared with the CON group, the HFHC diet group presented significant morphological abnormalities in hepatopancreatic cells, characterized by extensive cytoplasmic vacuolation and nuclear displacement. Supplementation with the three BAs markedly alleviated these pathological changes, including cytoplasmic vacuolation and nuclear displacement.

### Intestinal mucosal barrier-related gene expression

**Fig. 9:**
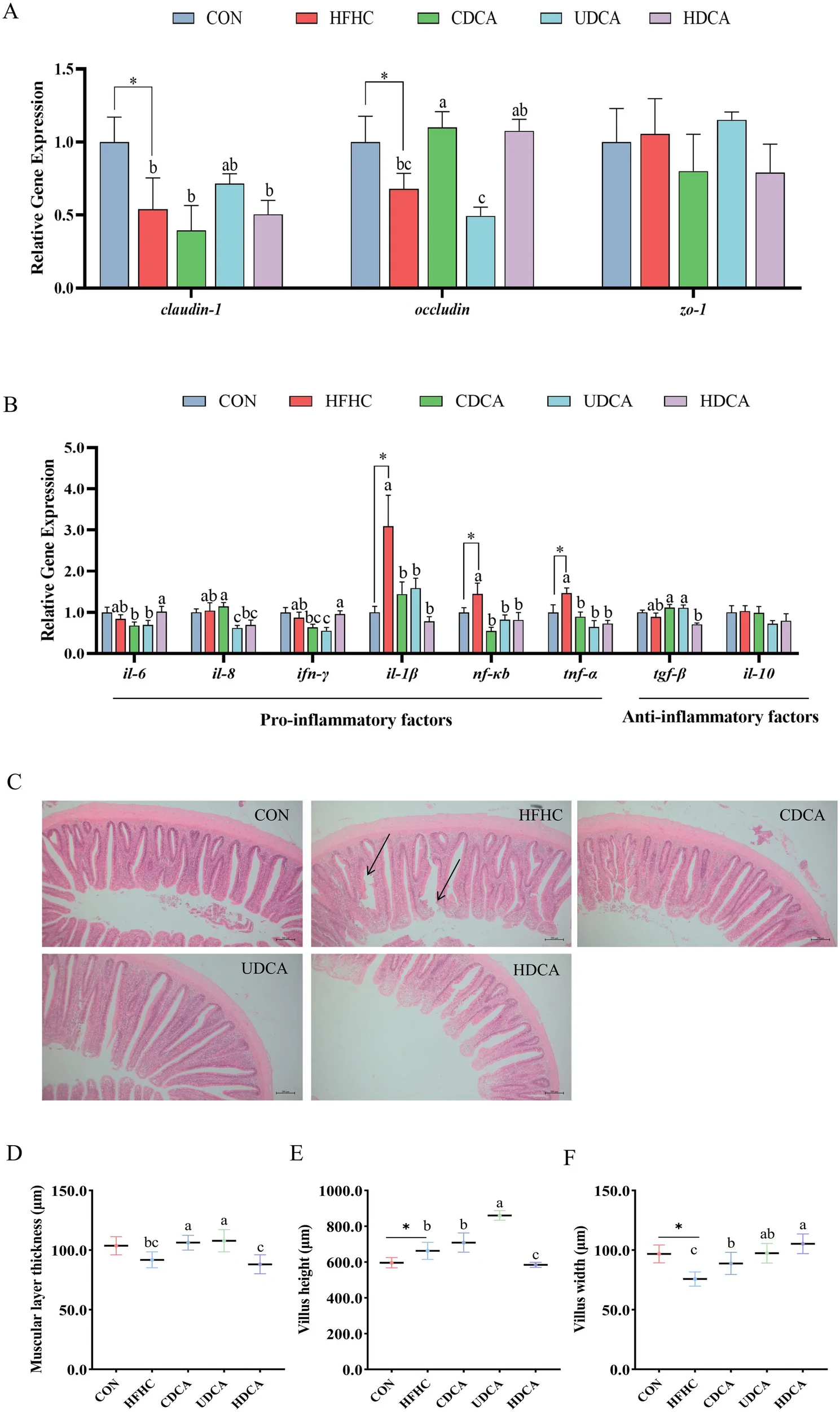
Effects of BAs on intestinal health of Yellow River carp. **A** The effects of BAs on the expression levels of intestinal mucosal barrier related genes in Yellow River carp, **B** The effects of BAs on the expression levels of intestinal inflammation related genes in Yellow River carp, **C** Intestinal H&E staining of Yellow River carp, **D** Intestinal muscular layer thickness, **E** Intestinal villus height, **F** Intestinal villus width. Data are presented as means ± S.E.M. The “*” indicates a significant difference between the control group and the HFHC group (*P* < 0.05). Different letters indicate significant differences between the HFHC group and the BAs group (*P* < 0.05)

The expression levels of genes associated with the intestinal mucosal barrier are presented in Fig. 9A. Compared with CON conditions, HFHC conditions significantly decreased *claudin-1* and *occludin* mRNA expression (*P* < 0.05). Compared with that in the HFHC group, the mRNA expression of *claudin-1* in the UDCA group trended to increase, while the mRNA expression of *occludin* in both the CDCA and HDCA groups was significantly greater than that in the HFHC group (*P* < 0.05).

### Intestinal inflammation-related gene expression

Intestinal inflammation-related gene expression was analyzed, and the results are depicted in Fig. 9B. Compared with the CON group, the HFHC group presented significantly upregulated *nf-κb*, *il-1β* and *tnf-α* mRNA expression (*P* < 0.05). Compared with those in the HFHC group, the mRNA expression of these three genes markedly decreased in all three bile acid groups (*P* < 0.05). Additionally, the mRNA expression of the pro-inflammatory cytokines *il-8* and *ifn-γ* was significantly reduced in the UDCA group (*P* < 0.05). However, no significant differences were observed in the mRNA expression of the anti-inflammatory factors *tgf-β* and *il-10* across all the experimental groups.

### Intestine histomorphology

HE staining was performed on intestinal tissue sections, followed by observation and analysis under a microscope. The results are presented in Fig. 9C-F. Compared with the CON group, the HFHC group presented a significant increase in intestinal villus height (*P* < 0.05) but a significant decrease in villus width (*P* < 0.05), with no significant difference in muscular layer thickness. Compared with the HFHC group, both the CDCA and UDCA groups presented significant increases in intestinal villus height, villus width, and muscular layer thickness (*P* < 0.05). The HDCA group presented a significantly greater villus width (*P* < 0.05), whereas no significant changes were detected in the villus height or muscular layer thickness.

### Effects of BAs on the species composition and diversity of the gut microbiota

**Fig. 10:**
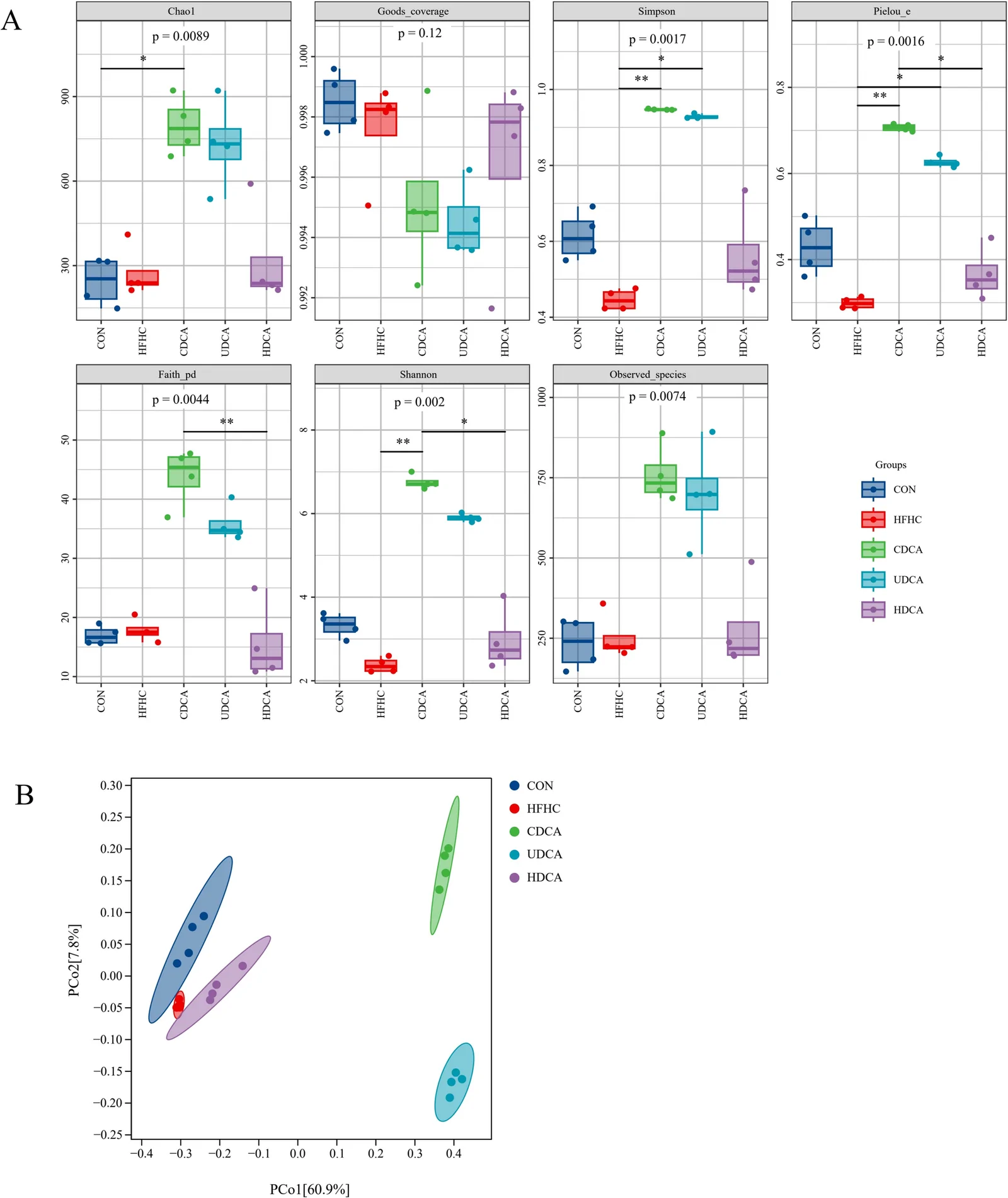
The effects of BAs on the intestinal microbial diversity in Yellow River carp. **A** Alpha diversity analysis, **B** PCoA

The alpha diversity parameters of the intestinal microbiome in the experimental fish were analyzed, and the results are shown in Fig. 10A. Specifically, CDCA supplementation significantly increased the Chao1, Simpson, Piclou_c, Faith_pd, and Shannon indices (*P* < 0.05). Moreover, UDCA significantly elevated Pielou’s evenness index (*P* < 0.05). Principal coordinates analysis (PCoA) further revealed significant differences in microbial community structure between the HFHC, CON, CDCA, and UDCA groups (Fig. 10B).

**Fig. 11:**
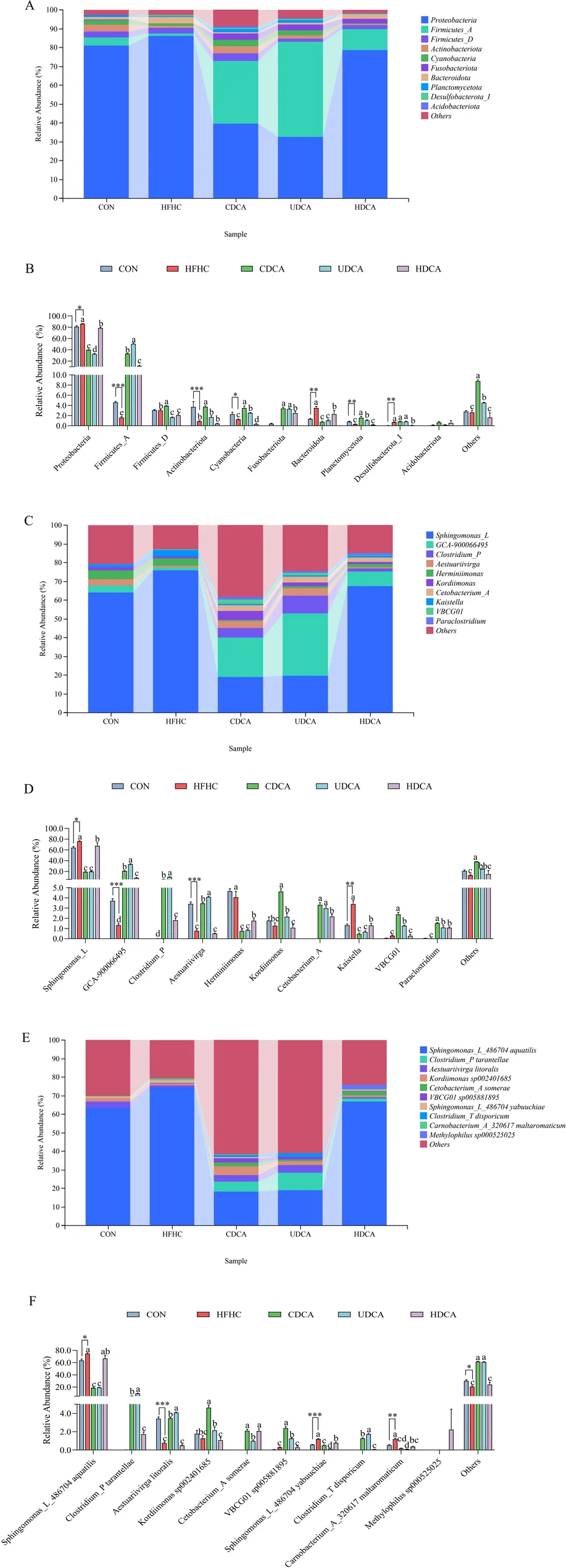
The effects of BAs on the gut microbial composition in Yellow River carp. **A** Relative abundance of intestinal microbiota at the phylum level, **B** Quantitative analysis of intestinal microbiota abundance at the phylum level. **C** Relative abundance of intestinal microbiota at the genus level, **D** Quantitative analysis of intestinal microbiota abundance at the genus level. **E** Relative abundance of intestinal microbiota at the species level, **F** Quantitative analysis of intestinal microbiota abundance at the species level. Data are presented as means ± S.E.M. The “*” indicates a significant difference between the control group and the HFHC group (*P* < 0.05). Different letters indicate significant differences between the HFHC group and the BAs group (*P* < 0.05)

This study characterized the intestinal microbial community structure of Yellow River carp. At the phylum level, *Proteobacteria*, *Firmicutes*, and *Actinobacteria* were identified as the dominant taxa (Fig. 11A, B). Relative to the CON group, the HFHC diet induced marked shifts in the intestinal bacterial community structure, as evidenced by the increase in *Proteobacteria* coupled with reduced abundances of *Firmicutes* and *Actinobacteria* (*P* < 0.05). CDCA and UDCA supplementation reversed HFHC-induced microbial dysbiosis by significantly suppressing *Proteobacteria* proliferation while enhancing the colonization of *Firmicutes* and *Actinobacteria*. Similarly, HDCA supplementation inhibited *Proteobacteria* growth, with a particularly pronounced increase in the relative abundance of *Firmicutes* within the microbial ecosystem.

At the genus level (Fig. 11C, D), compared with the CON diet, the HFHC diet increased the abundance of *Sphingomonas_L_486704 aquatilis* while decreasing the abundance of *Aestuariivirga* and *Herminiimonas*. Compared with the HFHC diet, all three bile acid interventions (CDCA, UDCA, and HDCA) markedly decreased *Sphingomonas* abundance while simultaneously promoting the expansion of beneficial genera including *Aestuariivirga*, and *Herminiimonas*.

Species-specific microbial analysis (Fig. 11E, F) demonstrated that HFHC diet feeding promoted *Sphingomonas* proliferation while simultaneously restricting *Aestuariivirga* colonization. In both the CDCA and UDCA treatment groups, the abundance of *Sphingomonas* significantly decreased, whereas the abundance of *Aestuariivirga* markedly increased. Moreover, bile acid supplementation resulted in substantial amplification of *Cetobacterium_A somerae* populations throughout the intestinal microbial community.

**Fig. 12:**
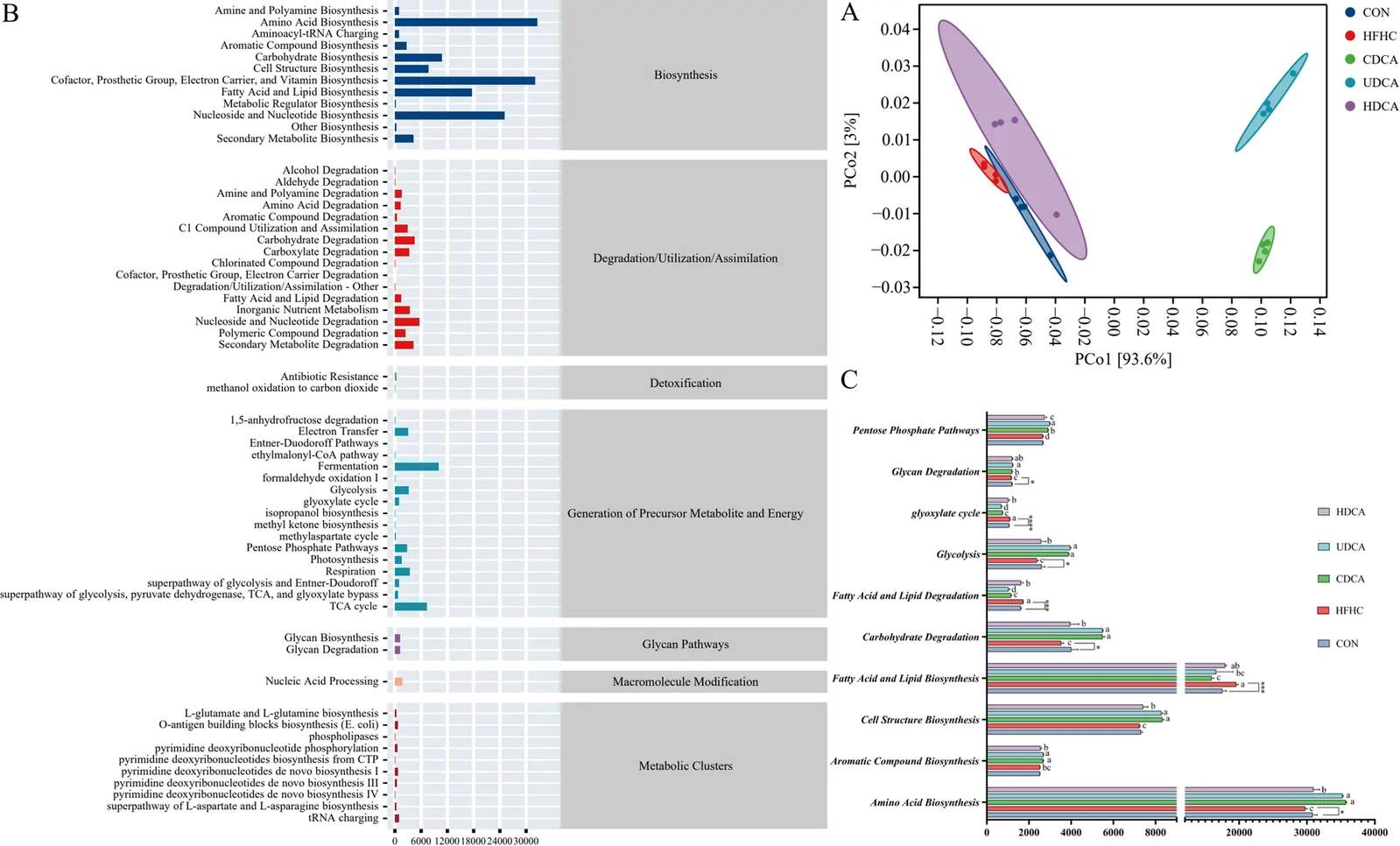
Effects of different BAs on the functional prediction of gut microbiota in Yellow River carp. **A** PCoA of the gut microbiota functional unit based on Bray-Curtis distances. **B** Predicted metabolic pathways of gut microbiota communities based on PICRUSt2 analysis. **C** Prediction and analysis of gut microbiota differential metabolic pathways

PCoA of functional units (Fig. 12A) revealed distinct clustering patterns. This finding indicates that at the functional unit level, significant differences existed between the HFHC group and the CON group, as did the bile acid groups. The KEGG pathway analysis results (Fig. 12B) highlighted the potential involvement of the gut microbiota in multiple biological processes. These processes include biosynthesis, degradation, assimilation, detoxification, generation of metabolic and energy precursors, glycan-related pathways, and macromolecular modifications. KEGG functional prediction analysis of metabolic pathways revealed that the fatty acid and lipid synthesis pathways associated with the gut microbiota in the HFHC group were significantly enriched. BAs supplementation induced marked changes in the functions of the gut microbiota, which were enriched predominantly in the pathways of amino acid biosynthesis, carbohydrate degradation, glycolysis, and cell structure synthesis (Fig. 12C).

## Discussion

HFHC diets have high energy density and can effectively improve fish growth efficiency, which are widely used in aquaculture [35–37]. The present study indicated that compared with the control group, HFHC significantly increased FBW, WGR and SGR of Yellow River carp and exerted an obvious growth-promoting effect. Notably, compared with the HFHC group, supplementation with UDCA and HDCA further significantly improved growth performance and effectively optimized the growth status of Yellow River carp under HFHC feeding conditions. As safe and highly efficient feed additives, BAs have demonstrated potential in promoting growth for various aquatic animals. Previous studies have confirmed that BAs can enhance the growth performance of fish. Specifically, dietary supplementation with BAs has improved the growth performance of *Channa argus* [38], *Oreochromis niloticus* [39], *Ctenopharyngodon idellus* [32], and *Larimichthys crocea* [33]. These findings indicate that BAs exert a positive effect on fish growth. The BAs used in these aforementioned studies were primarily commercial composite BAs with HDCA as the main component. Studies on the growth-promoting effects of single BAs have also been reported. For example, dietary supplementation with UDCA was shown to improve the growth performance of juvenile *Larimichthys crocea* [29]. The results of the present study demonstrate that adding BAs to a HFHC diet can significantly enhance the growth performance and feed utilization efficiency of Yellow River carp.

Serum lipids, glucose, and other hematological parameters can reflect the metabolic status of fish. In this study, significantly elevated serum TG and Glu levels were observed in Yellow River carp fed the HFHC diet, indicating that excessive carbohydrate and lipid intake induced glycolipid metabolic disorders. This phenomenon was consistent with findings in *Oncorhynchus mykiss* [40], *Dicentrarchus labrax* [41], and *Ctenopharyngodon idellus* [42]. Excessive cholesterol accumulation can cause cellular damage and is associated with the onset of metabolic diseases such as atherosclerosis and hepatic steatosis [43, 44]. In the present study, HFHC diet feeding led to a marked increase in the T-CHO levels in the hepatopancreas of Yellow River carp. However, dietary supplementation with UDCA significantly reduced T-CHO levels, whereas the levels of CDCA and HDCA also tended to decrease. This change was consistent with the elevated serum T-CHO content, suggesting that the HFHC diet might trigger lipid metabolism abnormalities in Yellow River carp and that BAs supplementation effectively alleviated such disorders. LDL-C plays a key role in the development of atherosclerosis, as it transports cholesterol from the liver to peripheral tissues. The addition of BAs mitigated this effect, reducing cholesterol accumulation and lowering LDL-C content. These findings are consistent with previous research on tiger puffer [30]. Our results showed that the HFHC diet caused cholesterol accumulation and a significant increase in LDL-C levels in the hepatopancreas. BAs supplementation mitigated hepatopancreatic cholesterol accumulation and decreased LDL-C levels accordingly, which was in line with findings in tiger puffer (*Takifugu rubripes*) [30]. The GOT and GPT are key indicators for evaluating hepatic inflammation. Upon hepatic injury, the release of these enzymes increases, resulting in elevated serum GOT and GPT levels [10, 45].The results of this study revealed that the HFHC diet can induce liver damage in Yellow River carp, as evidenced by significantly increased serum GOT and GPT levels. In contrast, BAs supplementation significantly reduced the serum levels of these two enzymes, demonstrating the hepatoprotective effects of BAs on the hepatopancreas.

Chronic consumption of a HFHC diet elevates free radical production, thereby inducing oxidative stress. This stress, stemming from an imbalance between reactive oxygen species (ROS) and antioxidant defense mechanisms, culminates in cell membrane damage, protein oxidation, and DNA impairment. Researchers have documented the potential of BAs to alleviate oxidative stress, with empirical evidence reported in various fish species. Dietary supplementation with BAs in a high-protein, high-fat diet for largemouth bass enhances antioxidant capacity and increases the activities of T-SOD, glutathione peroxidase (GSH-PX), and glutathione reductase (GR). This effect contributes to the reduction in excess oxygen free radicals generated during lipid peroxidation while simultaneously mitigating the deleterious effects of endotoxins and mycotoxins [46]. Furthermore, T-SOD play pivotal roles in ROS metabolism, thus reflecting an organism’s capacity to scavenge oxygen free radicals [47]. In the present study, compared with the HFHC diet, dietary supplementation with the three BAs significantly increased T-SOD activities in the hepatopancreas, decreased MDA levels, and increased the antioxidant capacity of Yellow River carp. In conclusion, the HFHC diet induced oxidative stress and compromised the antioxidant capacity of Yellow River carp, whereas supplementation with specific BAs markedly ameliorated these deleterious effects.

FXR, the primary receptor for BAs, is highly expressed in the liver and intestine. By interacting with the SHP signaling pathway, FXR regulates BAs metabolism, maintains its physiological concentration in the organism, and participates in the modulation of lipid and glucose metabolism [48–50]. Previous studies have also demonstrated that D-glucose can promote FXR gene expression [51]. In this study, the HFHC diet promoted *fxr* gene expression in the Yellow River carp hepatopancreas. Research has indicated that different BAs exhibit distinct binding affinities for FXR [52]. In this study, CDCA and HDCA significantly upregulated *fxr* gene expression, whereas UDCA increased the expression of the intestinal *tgr5* and *fgf19* genes. Studies have confirmed that CYP7A1 and CYP8B1 are key enzymes governing BA synthesis [53]. In this study, activated FXR protein promoted *shp* gene expression, which subsequently inhibited *cyp7a1* and *cyp8b1* gene expression. Studies have demonstrated that CDCA acts as a potent agonist of FXR [54, 55]. It regulates glucose and lipid metabolism mainly via the FXR/SHP pathway, and is readily converted to lithocholic acid by 7α-dehydroxylase of intestinal microbiota, which is also a strong FXR agonist. HDCA exhibits weaker FXR-activating activity than CDCA [56]. It is primarily modified by intestinal microbiota through 6α-hydroxylation to produce metabolites such as hyocholic acid. Accumulating evidence indicates that UDCA is a potent agonist of intestinal TGR5 [57]. With high stability in the intestinal tract, it mainly functions to protect the intestine and reduce glucose absorption. Therefore, such differences in receptor binding specificity may ultimately lead to functional divergence of the three bile acids in alleviating hepatointestinal damage in Yellow River carp. In conclusion, CDCA and HDCA significantly regulate BA metabolism in Yellow River carp associated with the FXR/SHP pathway. In contrast, UDCA can activate the intestinal TGR5 signaling pathway.

High-fat or high-carbohydrate diets promote gluconeogenic processes by increasing the activities of the key gluconeogenic enzymes G6Pase and PEPCK in the hepatopancreas of sea bass and common carp [34, 58]. In the present study, compared with the CON group, the HFHC group presented significantly upregulated expression levels and enzymatic activities of PEPCK and G6Pase, which contributed to the elevated serum glucose levels in Yellow River carp fed the HFHC diet. BA metabolism can modulate systemic glucose and lipid metabolism. Evidence indicates that increased *fxr* gene expression stimulates *shp* gene transcription, and *shp* then interacts with the transcription factors *foxo1* and *hnf4* to suppress the expression of their downstream target genes (*g6pase*, *pepck* and *fbpase*), thereby inhibiting gluconeogenesis [59]. In this study, the expression levels of *fxr* and *shp* in the hepatopancreas of Yellow River carp were increased in the CDCA and HDCA groups, whereas the expression of *foxo1* and *hnf4* was suppressed. Similarly, the enzymatic activities and gene expression levels of PEPCK and G6Pase were significantly inhibited in these two groups. These findings were further validated by protein immunofluorescence staining. Taken together, these results suggest that CDCA and HDCA may inhibit gluconeogenesis associated with the FXR signaling pathway, preventing the conversion of non-carbohydrate substrates into glucose and thus ameliorating hepatopancreatic glucose metabolic disorders induced by the HFHC diet.

Studies have demonstrated that BAs play a pivotal role in lipid metabolism. In the liver, FXR can induce increased expression of the *shp* gene, which in turn suppresses the expression of *srebp-1c* and its downstream hepatic lipogenic genes, thereby reducing hepatic lipid synthesis. In addition, FXR can upregulate *pparα* gene expression to increase fatty acid oxidation [22, 23]. In the present study, CDCA and HDCA suppressed the expression of the *srebp-1c* and *fas* genes while upregulating *pparα* gene expression associated with the FXR pathway. This regulatory effect reduced lipid synthesis and enhanced fatty acid β-oxidation in the hepatopancreas. This finding is consistent with the finding that the hepatopancreatic crude lipid content in the BAs-supplemented groups was lower than that in the HFHC group.

Excessive dietary carbohydrates and lipids significantly alter the gut microbiota structure and function, reducing beneficial bacteria and increasing pathogenic bacteria. This dysbiosis increases intestinal permeability, facilitating the translocation of bacteria and their metabolites across the intestinal barrier, and ultimately triggering inflammation [38]. Studies in *Mylopharyngodon piceus* and *Micropterus salmoides* have demonstrated that a high carbohydrate diet can lead to an increase in the mRNA expression levels of the pro-inflammatory factors *nf-κb*, *il-1β*, *tnf-α* and *il-6* [60, 61]. In addition, a high-fat and high-carbohydrate diet increases the permeability of intestinal epithelial cells and compromises the integrity of the intestinal mucosa. The intestinal mucosa, which serves as the first line of defense between the gut and the external environment, has been found to exhibit disrupted barrier function when associated genes are aberrantly expressed. This manifests as down regulation of tight junction proteins such as Claudin-1 and Occludin, leading to decreased intercellular connections and increased epithelial apoptosis. These alterations increase intestinal permeability, facilitating the translocation of bacteria, antigens, and endotoxins from the gut lumen into the mucosal lamina propria. This in turn, activates immune responses and triggers inflammation. In the present study, the HFHC group presented significantly greater intestinal mRNA expression of *nf-κb*, *il-1β* and *tnf-α*, and lower expression of *claudin-1* and *occludin* than the CON group did, indicating impaired intestinal mucosal structure. Overall, the HFHC diet induces intestinal inflammation in Yellow River carp by disrupting mucosal integrity and activating the NF-κB pathway. BAs regulate inflammatory responses via multiple mechanisms: they inhibit the NF-κB pathway to reduce pro-inflammatory cytokine expression, attenuate inflammation, and strengthen intestinal epithelial tight junctions to improve mucosal integrity, thus reducing bacterial translocation [38, 62]. In this study, supplemention with the three BAs in the HFHC diet significantly downregulated the mRNA expression of proinflammatory factors. With respect to tight junction genes, the CDCA and UDCA groups presented upregulated *occludin* mRNA expression. In summary, all three BAs alleviate intestinal inflammation by modulating the NF-κB pathway and pro-inflammatory factors, while CDCA and UDCA further protect the intestinal barrier by increasing tight junction protein expression.

Research on aquatic species has shown that BAs significantly influence the architecture and diversit of the gut microbiota y, reducing potentially harmful bacterial populations while promoting intestinal health [63]. The dietary inclusion of BAs has been shown to increase *Firmicutes* proportions and simultaneously decrease *Clostridium* levels within the intestinal microbial community of grass carp [64]. In sea bass, BAs enhance intestinal health by modulating microbial communities, promoting beneficial bacteria, and inhibiting harmful bacteria [58]. In this study, high-fat and high-carbohydrate diets altered the intestinal microbiota of Yellow River carp, increasing the abundance of *Proteobacteria* and *Sphingomonas_L_486704 aquatilis* while decreasing the abundance of *Firmicutes*. Bile acid supplementation reduced *Proteobacteria* and *Sphingomonas* but significantly increased *Firmicutes*. At the species level, it also increased *Cetobacterium_ somerae*, which has been shown to enhance the intestinal and hepatic health of Yellow River carp and reduce hepatic fat deposition [65]. BAs play a pivotal role in maintaining intestinal epithelial integrity and orchestrating glucose and lipid homeostasis in teleosts through the modulation of the gut microbial composition and metabolic activity.

## Conclusion

In conclusion, a HFHC diet induces dysregulated blood glucose and lipid homeostasis, excessive fat deposition in the hepatopancreas, and intestinal structural damage and functional impairment in Yellow River carp. Notably, BAs exhibit dual regulatory functions in Yellow River carp. On one hand, they ameliorate HFHC diet-induced glycolipid metabolic disorders. On the other hand, they maintain intestinal health by preserving intestinal morphology, alleviating inflammation and reshaping gut microbiota. From a practical perspective, these findings support the potential application of BAs as safe and effective feed additives to optimize fat- and carbohydrate-rich aquafeeds, which is conducive to improving the sustainability of intensive Yellow River carp farming.

## Supplementary Information

Supplementary Material 1.

## Data Availability

Sequence data that support the findings of this study have been deposited in the NCBI SRA database with the BioProject PRJNA1463531.

## Abbreviations

ANOVA: One-way analysis of variance
BA: Bile acid
CDCA: Chenodeoxycholic acid
CF: Condition factor
CREB: CAMP response element-binding protein
CYP7A1: Cholesterol 7α-hydroxylase
CYP8B1: Cholesterol 12α-hydroxylase
DAPI: 4’,6-diamidino-2-phenylindole
FAS: Fatty acid synthase
FBW: Final body weight
FE: Feed efficiency
FBPase: Fructose-1,6-bisphosphatase
FXR: Farnesoid X receptor
G6Pase: Glucose-6-phosphatase
GOT: Glutamic-oxaloacetic transaminase
GPT: Glutamic-pyruvic transaminase
GR: Glutathione reductase
GSH-PX: Glutathione peroxidase
HDCA: Hyodeoxycholic acid
HDL-C: High-density lipoprotein cholesterol
H2O2: Hydrogen peroxide
HSI: Hepatic somatic index
IBW: Initial body weight
LDL-C: Low-density lipoprotein cholesterol
MDA: Malondialdehyde
NF-κB: Nuclear factor κB
PCoA: Principal coordinate analysis
PEPCK: Phosphoenolpyruvate carboxykinase
PPARα: Peroxisome proliferator-activated receptorα
S.E.M.: Standard error of the mean
SGR: Specific growth rate
SHP: Small heterodimer partner
SR: Survival rate
SREBP: Sterol regulatory element-binding protein
T-CHO: Total cholesterol
TG: Triglycerides
TGR5: Takeda G protein-coupled receptor 5
T-SOD: Total superoxide dismutase
UDCA: Ursodeoxycholic acid
VSI: Visceral index
WGR: Weight gain rate
