# Supplementary material for: Dual regulation of bile acids: ameliorating hepatic glycolipid disorders and restoring intestinal health in HFHC-challenged Yellow River carp (Cyprinus carpio L.)
Source: BMC Vet Res. 2026 Jul 7;22:575. doi: 10.1186/s12917-026-05688-1 (PMC13637175; doi:10.1186/s12917-026-05688-1)
Supplement: Supplementary file 1 [file 12917_2026_5688_MOESM1_ESM.docx]

**Table S1.** Diet formula and nutritional composition (%).

| **Component (%)** | **Diets** | | | | |
| --- | --- | --- | --- | --- | --- |
|  | **CON** | **HFHC** | **CDCA** | **UDCA** | **HDCA** |
| Fishmeal | 15.00 | 15.00 | 15.00 | 15.00 | 15.00 |
| Corn protein powder | 17.00 | 17.00 | 17.00 | 17.00 | 17.00 |
| Soybean meal | 21.00 | 21.00 | 21.00 | 21.00 | 21.00 |
| Rapeseed cake | 4.00 | 4.00 | 4.00 | 4.00 | 4.00 |
| Wheat meal | 9.00 | 9.00 | 9.00 | 9.00 | 9.00 |
| Com starch | 11.00 | 23.00 | 23.00 | 23.00 | 23.00 |
| Soybean oil | 2.50 | 8.40 | 8.40 | 8.40 | 8.40 |
| Ca(H_2_PO_4_)_2_ | 1.00 | 1.00 | 1.00 | 1.00 | 1.00 |
| Vitamin Premix | 0.07 | 0.07 | 0.07 | 0.07 | 0.07 |
| Mineral Premix | 0.10 | 0.10 | 0.10 | 0.10 | 0.10 |
| Bile acid | 0.00 | 0.00 | 0.03 | 0.03 | 0.03 |
| Choline chloride | 0.20 | 0.20 | 0.20 | 0.20 | 0.20 |
| Carboxy methyl cellulose | 1.00 | 1.00 | 1.00 | 1.00 | 1.00 |
| Microciystalline cellulose | 18.13 | 0.23 | 0.20 | 0.20 | 0.20 |
| Total | 100.00 | 100.00 | 100.00 | 100.00 | 100.00 |
| Proximate composition (%) |  |  |  |  |  |
| Crude protein | 30.60 | 30.35 | 30.82 | 30.49 | 30.61 |
| Crude lipid | 5.57 | 11.28 | 11.11 | 11.18 | 11.23 |
| Crude ash | 5.57 | 5.44 | 5.14 | 4.35 | 4.74 |

Note: 1. Containing the following per kilogram of vitamin premix: VA 2000000~6000000 IU, VD3 1000000~2000000 IU, VE 9000~30000 mg, VK3 2700~9000 mg, VB1 550~1800 mg, VB2 860~2880 mg, VB6 5500~18000 mg, VB12 10~20 mg, nicotinamide 10000~42000 mg, folic acid 900~3000 mg, calcium pantothenate 5500~18000 mg, biotin 20~60 mg, VC 6000~20000 mg, inositol 7200~24000 mg. 2. Containing the following per kilogram of mineral premix: Cu 3000~12000 mg, Fe 5000~20000 mg, Zn 8000~32000 mg, Mn 10000~40000 mg, I 200~800 mg, Se 100~400 mg.

**Table S2.** Primer sequences of target genes in real-time PCR analysis.

| **Target gene** | **Primer sequence (5′-3′)** | **GenBank ID** |
| --- | --- | --- |
| *18s* | F: GAGACTCCGGCTTGCTAAAT  R: CAGACCTGTTATTGCTCCATCT | LN591197.1 |
| *fxr* | F: CCCCTACAGCCATCAGT  R: GACCGACAATGCTCCCT | XM_042729372.1 |
| *shp* | F: GAAGTTCTGGAGTCTGGATT  R: GGATGAAGAGGCACAAGG | XM_042741839.1 |
| *cyp7a1* | F: CTACCTGACCCTCTTTGG  R: CGTGGAGCATTGTCTTGG | XM_042718130.1 |
| *cyp8b1* | F: CTTGCCATTGCTTGTCAT  R: GTATTCGGCTATCGTTCC | XM_019073965.2 |
| *fgfr4* | F: CTACGCAACTTCACTATCACA  R: CCAGCATCATCAAGACCATT | XM_042747649.1 |
| *tgr5* | F: GCATTACGAACGCTACCTGTG  R: TGGAAGAGTTACGAGATGTGGT | XM_042764258.1 |
| *foxo1* | F: ACGGCTCACCTTATCTCA  R: CTTGCTGTTGTTGTCCATAG | XM_042739020.1 |
| *hnf4* | F: CAGGATTAGCACCAGAAGAT  R: TGTCGCCATTCAAGATAGG | XM_019071428.2 |
| *pgc-1α* | F: TGCTGCTTTGGTTGGTGAAG  R: TGCATCGAGGTCACTGACATC | AB767302.1 |
| *fbpase* | F: CCTGCCATCGGTGAGTTTAT  R: CCATCCTCTGGGAATTTCTTCT | AF427864.1 |
| *pepck* | F: ACTCTTTGGGCAGACCTTTAC  R: CTGTCTGGTGTCAGGAAGATG | KP250869.1 |
| *g6pase* | F: GAGGCCTTCAACAGACAGAAA  R: GAGCTTTGAGAAGCAGGTACAA | AF427863.1 |
| *gys* | F: TTTACTACACATGCCACCCTAC  R: TCTCTCCTGCCTCTTGATCTAT | XM_019090903.1 |
| *gsk-3β* | F: ATCAGGTGCATGAGGAGTTGAG  R: AGCAGAGCATTTCCAAACGC | XM_042774357.1 |
| *creb* | F: GAGACAGACACTCAGCAGATTG  R: TGGATGACGCCGTGAACTT | XM_019107844.2 |
| *pk* | F: GATATGGGCTCTGCCTTCAT  R: TCCGATGGTGCAGATGATTC | XM_042744551.1 |
| *hk* | F: CGTTGTAGCCGTAGTGAATGA  R: GCTACCCGTTCCTGCAATTA | XM_019097065.2 |
| *srebp-1c* | F: CGTCTGCTTCACTTCACTACTC  R: GGACCAGTCTTCATCCACAAA | XM_042730308.1 |
| *fas* | F: GACAGGCCGCTATTGCTATT  R: TGCCGTAAGCTGAGGAAATC | GQ466045.1 |
| *cpt-1* | F: CAGATGGAAAGTGTTGCTAATGAC  R: TGTGTAGAAGTTGCTGTTGACCA | JQ361077.1 |
| *pparα* | F: GGGAAAGAGCAGCACGAG  R: GCGTGCTTTGGCTTTGTT | FJ849065.1 |
| *fatp* | F: AATCAGCCGAAGAAGGACAC  R: AATCCACAGGCACCCGTCTT | XM_019081506.1 |
| *glp-1α* | F: TCACGCTGAGGGAACCTA  R: GCTTGGGCTGTCCAGATT | XM_019094634.2 |
| *fgf19* | F: GTGGGAAAACTGTATGGATCGC  R: AAGGGCTCCATGTTTGCTTG | XM_042728520.1 |
| *glut2* | F: GAGGGTCTTTGTGGGAACTATG  R: GTTTCAGGTACACGCAAGTAGA | XM_042718276.1 |
| *sglt1* | F: CTAAAGAAGAGGAGGCAGAGTTG  R: ACAGACGGTGAGGAGGATAATA | JN867793.1 |
| *claudin-1* | F: CTGGAGTTGATGGGTTTCTTTCTTTTG  R: AGACCTTTCATGCTTTCTACCG | XM_042749400.1 |
| *occludin* | F: CAGGAGGCATCCATGGTGTT  R: AAGACACTGCCGGTCTTCAG | XM_042729457.1 |
| *zo-1* | F: CCGAAGCTTTGACAGCAAAC  R: GGTTGATCTTCCACTGACTC | XM_0427600882.1 |
